# Functional and Transcriptome Analysis Reveal Specific Roles of *Dimocarpus longan DlRan3A* and *DlRan3B* in Root Hair Development, Reproductive Growth, and Stress Tolerance

**DOI:** 10.3390/plants13040480

**Published:** 2024-02-07

**Authors:** Qilin Tian, Xiying Xie, Ruilian Lai, Chunzhen Cheng, Zihao Zhang, Yukun Chen, Xu XuHan, Yuling Lin, Zhongxiong Lai

**Affiliations:** 1 Institute of Horticultural Biotechnology, Fujian Agriculture and Forestry University, Fuzhou 350002, China; tianql70@163.com (Q.T.); xxiying@foxmail.com (X.X.); lairuilian@faas.cn (R.L.); ld0532cheng@126.com (C.C.); zhangzihao863@163.com (Z.Z.); cyk68@163.com (Y.C.); xxuhan@163.com (X.X.); buliang84@163.com (Y.L.); 2 School of Media and Design, Nantong Institute of Technology, Nantong 226019, China; 3 Institut de la Recherche Interdisciplinaire de Toulouse, IRIT-ARI, 31300 Toulouse, France

**Keywords:** *Dimocarpus longan* Lour., ras-related nuclear protein (Ran), promoter, root hair, reproduction, stress responses, transcriptome analysis

## Abstract

Ran GTPases play essential roles in plant growth and development. Our previous studies revealed the nuclear localization of DlRan3A and DlRan3B proteins and proposed their functional redundancy and distinction in *Dimocarpus longan* somatic embryogenesis, hormone, and abiotic stress responses. To further explore the possible roles of *DlRan3A* and *DlRan3B*, gene expression analysis by qPCR showed that their transcripts were both more abundant in the early embryo and pulp in longan. Heterologous expression of *DlRan3A* driven by its own previously cloned promoter led to stunted growth, increased root hair density, abnormal fruits, bigger seeds, and enhanced abiotic stress tolerance. Conversely, constitutive promoter *CaMV 35S* (*35S*)-driven expression of *DlRan3A*, *35S*, or *DlRan3B* promoter-controlled expression of *DlRan3B* did not induce the alterations in growth phenotype, while they rendered different hypersensitivities to abiotic stresses. Based on the transcriptome profiling of longan *Ran* overexpression in tobacco plants, we propose new mechanisms of the *Ran*-mediated regulation of genes associated with cell wall biosynthesis and expansion. Also, the transgenic plants expressing *DlRan3A* or *DlRan3B* genes controlled by *35S* or by their own promoter all exhibited altered mRNA levels of stress-related and transcription factor genes. Moreover, *DlRan3A* overexpressors were more tolerant to salinity, osmotic, and heat stresses, accompanied by upregulation of oxidation-related genes, possibly involving the Ran-RBOH-CIPK network. Analysis of a subset of selected genes from the *Ran* transcriptome identified possible cold stress-related roles of brassinosteroid (BR)-responsive genes. The marked presence of genes related to cell wall biosynthesis and expansion, hormone, and defense responses highlighted their close regulatory association with *Ran*.

## 1. Introduction

The longan tree (*Dimocarpus longan* Lour., Sapindaceae), a tropical evergreen fruit tree in southern China, is of great edible value and exhibits medicinal properties. However, there are certain challenges such as lacking aborted-seed varieties and the influence of environmental stresses on vegetative growth, fruit yield, and quality [1]. Being a Sapindaceae family of fruit crops, thicker arils and abortive seeds are important quality traits. In recent years, therefore, there have been several reports on the mechanisms of longan fruit and seed development which are controlled by various regulators [2,3].

Ras-related nuclear protein (Ran) GTPases, also known as molecular switches, play a universal role in nucleocytoplasmic transport, specifically expressed during embryogenesis, and are essential for cell division in animal development [4,5]. Plant Ran GTPases, along with their cofactors and nucleoporins, are involved in diverse biological processes including plant growth and root development [6,7], regulation of hormone (such as auxin and abscisic acid, ABA) sensitivity [6,8,9], enhancement of disease resistance [10], and stress responses, such as those to drought, salinity, osmotic stress, and cold and oxidative stresses [7,8,11]. This indicates that plant *Ran* may have a remarkable impact on plant growth and, hence, its economic traits when overexpressed.

Previous studies by our research group have cloned *DlRan3A* and *DlRan3B*, two *Ran* genes from somatic embryos in *D. longan*, and revealed their involvement in longan somatic embryogenesis [12]. *DlRan3A* and *DlRan3B* promoters (hereafter, referred to as ‘p*DlRan3A*’ and ‘p*DlRan3B*’, respectively), 1256 bp and 1569 bp in length, were cloned from embryogenic callus (EC) [13,14]. The deletion experiments showed that p*DlRan3A* and p*DlRan3B* might be involved in transcriptional control of plant hormones and specific defense reactions [14,15]. However, the molecular mechanism of longan *Ran* and its relationship with plant growth (especially seed and fruit development) and responses to the external environment need further investigation. 

In this study, expression profiling of *DlRan3A* and *DlRan3B* was analyzed in different longan tissues, during zygotic embryo development, and in the pulp of ripening fruits, to reveal the potential role of *Ran* in embryo and fruit development in woody fruit trees. Currently, the molecular basis of the *Ran* gene and its promoter for the regulation in plant embryo and fruit development is poorly understood, in part due to the fact that few studies have aimed to investigate global changes in the gene expression elicited by overexpression of the plant *Ran* gene. For selection and further application of *Ran* genes in molecular breeding using transgenic technology, we generated transgenic tobacco plants overexpressing *DlRan3A* and *DlRan3B*, using both *CaMV 35S* (*35S*) and their own promoters. Based on the analysis of plant phenotype and environmental stress response, transcriptome analysis of transgenic lines was subsequently performed to identify the possible downstream genes responsible for the specific plant growth and stress tolerance phenotypes among different transgenic lines. The selected genes shared or were distinct between transgenic lines, respectively, will be useful to examine the effect and underlying molecular mechanism of longan *Ran* overexpression, and for genetic engineering to enhance the stress tolerance of woody fruit trees, including longan. 

## 2. Results

### 2.1. Longan DlRan3A and DRan3B Are Highly Expressed in Early Embryos and Pulp (Aril)

**Figure 1:**
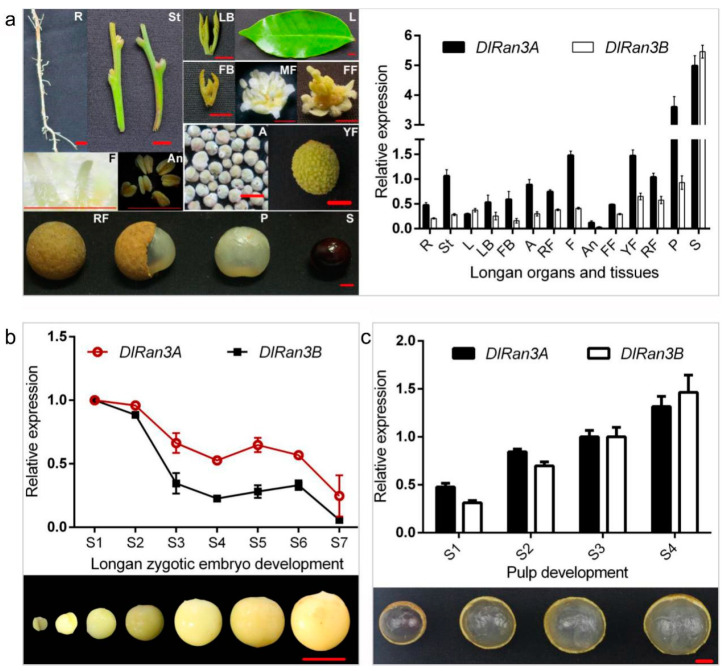
Relative expression levels of *DlRan3A* and *DlRan3B* in different tissues, developing zygotic embryos and pulp in longan. (**a**) Relative expression of *DlRan3A* and *DlRan3B* in different tissues (updated from Chen [16]). R: root; St: stem; L: leaf; LB: leaf bud; FB: floral bud; A: alabastrum; MF: male flower; F: filament; An: anther; FF: female flower; YF: young fruit; RF: ripe fruit; P: pulp; S: seed; bar = 5 mm. (**b**) Relative expression of *DlRan3A* and *DlRan3B* during zygotic embryo development. Longan zygotic embryos from stage S1 to S7 were collected from 16 June to 12 July in 2015, every four or every five days; bar = 10 mm. (**c**) Relative expression of *DlRan3A* and *DlRan3B* in different sizes of longan pulp. Longan pulp from stage S1 to S4 were collected in August 2015; bar = 10 mm.

To explore the potential developmental role of *DlRan3A* and *DlRan3B*, we first investigated their expression patterns in *D. longan* Lour. cv. Honghezi in diverse tissues (Figure 1a). We found that, apart from the fundamental expressions in longan tissues, *DlRan3A* and *DlRan3B* were both predominantly expressed in the seeds and then in the pulp, but displayed the lowest levels in the anthers (Figure 1a). This indicates that *Ran* is indispensable to longan growth and development while *DlRan3A* and *DlRan3B* genes might play more significant roles in seed and pulp development.

To further characterize *DlRan3A* and *DlRan3B* expression patterns during longan seed (embryo) and pulp development, we next examined their expressions during ‘Honghezi’ zygotic embryo development and in the pulp of ripening fruits. In the early stages (S1 and S2), both genes showed high expression with a fluctuating trend that declined during the zygotic embryo development (Figure 1b). However, both gene expressions showed an increase in the pulp of ripening fruits (Figure 1c). 

### 2.2. Characterization of DlRan3A and DlRan3B Genes and the Use of Their Own Promoters by Heterologous Expression in Tobacco Plants

To further investigate the function of *DlRan3A* and *DlRan3B*, we cloned each gene into pCAMBIA1301 driven by *35S* or *Ran* promoters and transformed into *Agrobacterium tumefaciens* strain EHA105. The T2 line *35S*-driven expression of *DlRan3A* or *DlRan3B* (henceforth, referred to as ‘35S_A’ or ‘35S_B’, respectively) and those driven by the *Ran* promoter (hereinafter, referred to as ‘PA_A’ or ‘PB_B’, respectively) were employed for the following experiments.

**Figure 2:**
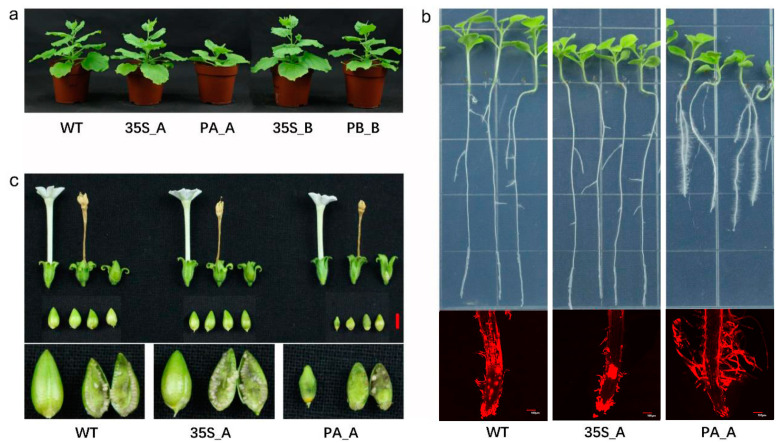
Phenotype of transgenic tobaccos. (**a**) The entire phenotypes of transgenic tobaccos. WT and T2 lines of P35S_A, PA_A, P35S_B, and PB_B (45 d) are displayed in the graph from left to right. (**b**) The phenotype of transgenic tobacco roots (21 d). WT and T2 lines of P35S_A and PA_A are displayed in the graph from left to right. The upper images illustrate the transgenic tobaccos germinated and vertically cultivated on MS medium for 21 d, and the lower images illustrate the 21-day-cultivated tobacco roots stained with propidium iodide, as observed under a confocal microscope (bar = 100 μm). (**c**) The phenotypes of flowers, fruits, and seeds of transgenic tobaccos. The WT and T2 lines of P35S_A and PA_A are displayed in the graph from left to right (bar = 5 mm).

At the same planting time, excluding PA_A, there were no significant differences in plant height and blade generation rate among the transgenic tobaccos (Figure S3). Notably, PA_A showed a dwarf phenotype with reduced height and thick stems, along with phenotypes of late flowering and reduced axillary buds (Figure 2a). After vertical cultivation for 7 d, 14 d, and 21 d, excluding PA_A, there were no significant differences in root growth among the transgenic tobaccos. Notably, PA_A exhibited a sturdier root system along with increased numbers of root hairs (Figure 2b and Figure S4). Even after 50–60 d of growth, PA_A maintained distinct phenotypes in fruit and seed development, showing abnormal fruits, larger seeds, decreased seed setting rate, and increased seed weight (Figure 2c and Figure S5). This indicates that p*DlRan3A*-driven expression of *DlRan3A* caused stunted growth and significantly affected the development of root, fruit, and seed. Moreover, the histochemical staining assay conducted to assess p*DlRan3A*- or p*DlRan3B*-driven expression of the *GUS* gene in transgenic tobaccos (hereafter, referred to as ‘PA_GUS’ or ‘PB_GUS’, respectively) revealed that both the promoters led to *GUS* expression in roots, leaves, flowers, fruits, and seeds. However, the *DlRan3A* promoter could drive greater *GUS* accumulation in petals (Figures S6 and S7).

### 2.3. Heterologous Expression in Tobaccos Reveals Shared and Distinct Roles of DlRan3A and DlRan3B in Various Stresses

**Figure 3:**
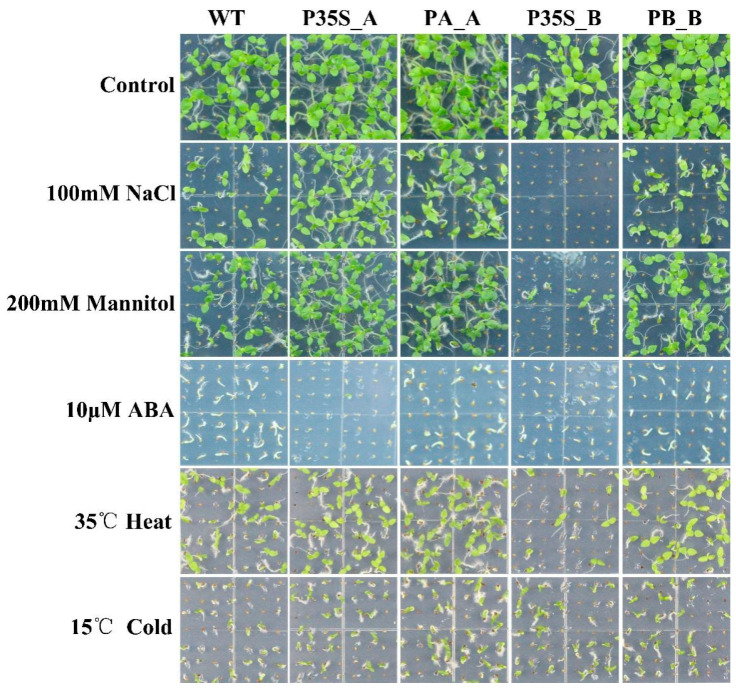
Abiotic stress tolerance of transgenic tobaccos. Graphics related to the WT and T2 lines of P35S_A, PA_A, P35S_B, and PB_B are displayed from left to right; a control group without any treatment and the treatment groups belonging to 100 mM NaCl, 200 mM mannitol, 10 μM ABA, 35 °C heat, and 15 °C cold stresses are displayed from top to bottom.

Under 100 mM NaCl stress, 200 mM mannitol stress, 10 μM ABA, 35 °C heat stress, and 15 °C cold stress, tobaccos expressing *DlRan3A* (i.e., P35S_A and PA_A) exhibited stronger tolerance to salinity, osmotic, and heat stresses. In contrast, tobaccos expressing *DlRan3B* (i.e., P35S_B, and PB_B) showed relatively weaker tolerance against abiotic stress. Remarkably, P35S_B tobaccos were hypersensitive to salinity, osmotic, and heat stresses, while P35S_A tobaccos were hypersensitive to ABA. Additionally, tobaccos expressing *DlRan3A* or *DlRan3B* showed mild tolerance against cold stress (Figure 3).

### 2.4. Transcriptome Profiling of Transgenic Tobaccos Revealed Alteration in Genes Involved in Biosynthetic Processes Related to the Cell Wall, Hormone Signaling, and Stress Responses

To understand the molecular functions of longan *Ran* GTPases, RNA-sequencing (RNA-Seq) profiling was conducted to estimate the effects on the entire transcriptome. Sequencing libraries were generated from WT, P35S_A, P35S_B, PA_A, and PB_B transgenic tobacco plants cultivated in Murashige and Skoog (MS) medium for 21 d. Reads alignment to the reference genome is shown in Table S5. The differentially expressed genes (DEGs) with higher transcript levels in transgenic lines than the corresponding control sample were denoted “up-regulated genes”, whereas those with lower transcript levels were defined as “down-regulated genes”. As shown in Table S6, the PB_B versus WT group (hereinafter, referred to as ‘PB_B/WT’) showed the largest number of DEGs, followed by PA_A versus WT groups (hereinafter, referred to as ‘PA_A/WT’); the DEGs in the PA_A versus P35S_A groups or PB_B versus P35S_B groups (hereinafter, referred to as ‘PA_A/P35S_A’ or ‘PB_B/P35S_B’, respectively) were less than any transgenic lines versus the WT groups.

Notably, not many DEGs in P35S_A versus WT groups (hereinafter, referred to as ‘35S_A/WT’) were shared with the P35S_B versus WT groups (hereinafter, referred to as ‘35S_B/WT’) (Figure S8a and Table S7). Furthermore, functional annotation revealed that the shared DEGs are mainly involved in cell wall organization or biogenesis (such as cellulose synthase, CESA) and plant resistance (such as asparagine synthetase, AS, and heat shock protein, HSP). Importantly, PA_A/WT and 35S_A/WT shared a certain number of DEGs (Figure S8b and Table S8). These are also mainly involved in cell wall organization or biogenesis (such as cellulose synthase) and plant resistance, such as AS, HSP, pleiotropic drug resistance protein (PDR), calmodulin-binding protein (CBP), peroxide (PER), and respiratory burst oxidase homolog (RBOH). Among the DEGs, most DEGs were up-regulated, except some like *CYP450* (cytochrome P450) and *HSP*. The shared DEGs between PB_B/WT and 35S_B/WT are listed in Figure S8c and Table S9, including calcium-binding protein, CESA, expansin, and genes involved in plant resistance (such as *AS*, *NAC*, *ERF*, *WRKY*, *bHLH*, *C3H*, *GRAS*, and *HSP*). Among these, most DEGs associated with plant resistance were down-regulated. 

Additionally, we also analyzed the expression profiles of transcription factor genes, cell wall biosynthesis and expansin/extensin genes, hormone-related genes, and stress-related genes between non-transgenic and transgenic tobaccos. As shown in Figure S9, the heat map indicated that certain transcription factors (MYB, NAC, WRKY, ERF, GRAS, bHLH, etc.) were down-regulated in the transgenic lines, especially in the 35S_B and PB_B lines. Regarding hormone-related genes, auxin-related DEGs were up- or down-regulated; ethylene-related *ERFs* were mostly down-regulated, especially in the 35S_B and PB_B lines. Also, all of the brassinosteroid (BR)-related *EXORDIUMs* were up-regulated in the transgenic lines, along with down-regulated *MYC2*s (jasmonic acid-related) and up-regulated *ARR9* (cytokinin-related). Notably, most extensin genes were up-regulated in PA_A along with three extensin genes (Niben101Scf02042g00001, Niben101Scf02191g03003, and Niben101Scf03036g00006). The two *expansins* (Niben101Scf03913g01045 and Niben101Scf20887g00008) were significantly up-regulated or down-regulated in the PA_A/P35S_A comparisons, respectively (Figure S10). The expression profiles of stress-related genes are shown in Figures S11 and S12.

### 2.5. Functional Classification, and Kyoto Encyclopedia of Genes and Genomes (KEGG) Analyses of DEGs

Gene Ontology (GO) enrichment analysis was performed for global functional analysis of DEGs related to longan *Ran* expression. Compared to WT, PA_A showed the greatest number of significantly enriched pathways and related DEGs (Table S10). 

In P35S_A (compared to WT), the oxidoreductase activity pathway acting on peroxide as an acceptor was significantly enriched for four DEGs: the peroxide gene, *RBOH*, and Novel00358, were up-regulated, while *CAT1* (catalase isozyme 1) was down-regulated. In PA_A (compared to WT), among the sixteen significantly enriched pathways, “cell wall organization or biogenesis” or related pathways were predominant along with six up-regulated DEGs (mainly extensin gene). Other pathways were mainly enriched for upregulated *CESAs*, *XET*/*XTH* (xyloglucan endotransglucosylase/hydrolase), and *UGE* (Bifunctional UDP-glucose 4-epimerase and UDP-xylose 4-epimerase), while *UGT73C3* (UDP-glycosyltransferase 73C3), *GT* (Anthocyanidin 3-O-glucosyltransferase), and *CIPK* (Calcineurin B-like-interacting serine/threonine-protein kinase 11) were down-regulated.

Further comparative analysis of PA_A/P35S_A or PB_B/P35S_B illuminated the biological function of longan *Ran* promoters. For PA_A/P35S_A or PB_B/P35S_B, all the listed cell wall biogenesis pathways were significantly enriched for the three extensin genes mentioned above. However, these were up-regulated in PA_A/P35S_A but down-regulated in PB_B/P35S_B.

Likewise, in PA_A/PB_B, the significantly enriched pathways were mainly related to cell wall and oxidative stress. Notably, the oxidative stress-related pathways were significantly enriched for four up-regulated peroxide genes (Niben101Scf03990g00010, Niben101Scf00416g01009, Niben101Scf02709g01005, and Niben101Scf02349g03001) and a *GPX* (glutathione peroxidase) gene (Niben101Scf06369g03006) (also see Figure S13).

Moreover, the KEGG biochemical pathways analysis of related DEGs was implemented. Compared to WT, the pathways related to alanine, aspartate, and glutamate metabolism were significantly enriched in P35S_A, P35S_B, or PA_A, lines, respectively (Table S11), suggesting the key role of longan *Ran* in these processes. Also, we found five significantly enriched pathways in PA_A (compared to PB_B). Most of these pathways were related to up-regulated DEGs, including five *GST* (glutathione S-transferase) (Niben101Scf00069g02013, Niben101Scf10316g03004, Niben101Scf03147g10010, Niben101Scf03482g0101, and Niben101Scf11037g00006) and a *GPX* (mentioned above in the GO analysis of PA_A/PB_B) genes.

### 2.6. Verification qPCR Validation of Candidate DEGs

**Figure 4:**
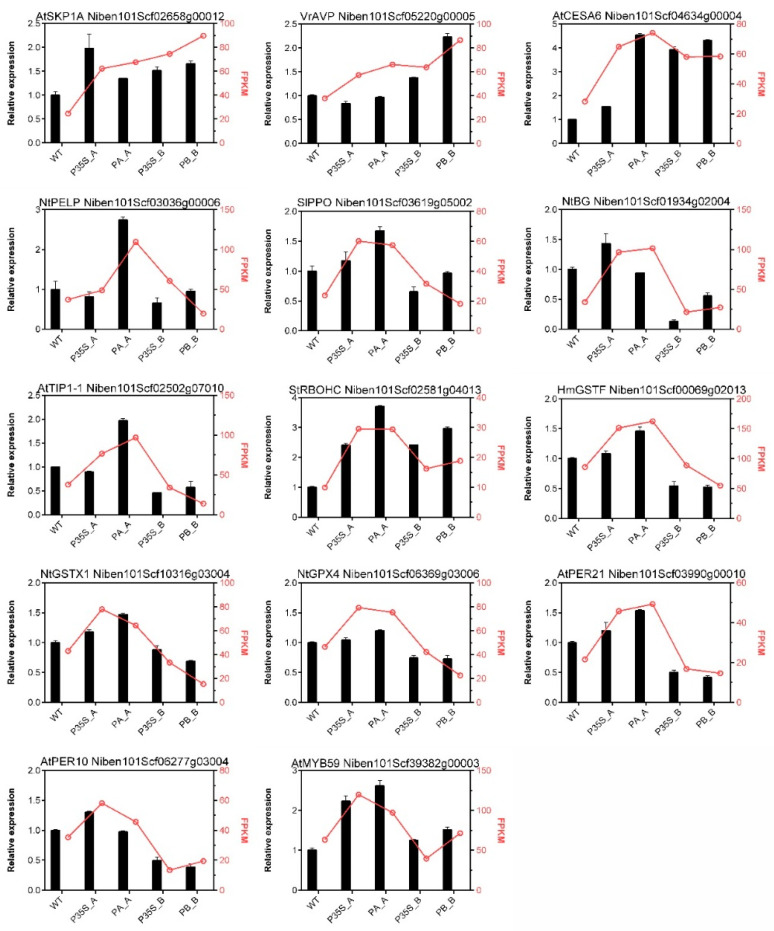
Validation of RNA-seq results by qPCR. The corresponding gene ID is mentioned at the top of each graph. The columns represent the relative expression levels measured by qPCR using *NbEF1a* as a reference gene, and the red lines portray the FPKM change from RNA-seq.

To validate the RNA-Seq results, 14 DEGs were selected for qPCR assay. The gene symbols, FPKM values, and relative expression levels are shown in Figure 4. Although the fold changes in transcript levels detected by RNA-Seq and qPCR were not perfectly matched, most of the candidate DEGs showed similar trends of change in gene expression as in the RNA-Seq results. In general, the expression patterns measured by the two methods were consistent.

## 3. Discussion

### 3.1. DlRan3A and DlRan3B Up-Regulate Cell Wall Biosynthesis and Expansion Genes to Regulate Early Embryo and Pulp Development in Longan

Studies have proved that plant Ran is ubiquitously expressed in all tissues, such as in tomato [17], Arabidopsis [18], tall fescue [19], sugarcane [20], etc. In longan, *DlRan3A* and *DlRan3B* also exhibited a certain fundamental expression in almost the whole tree, suggesting their essential roles in development. *Ran* is more expressed in meristematic tissues, embryos, or roots, which provides a clue to its functional diversity in plants [18,19,20]. Arabidopsis RAN1 interaction with DEM1 (defective embryo and meristems 1) is required for organized cell divisions during embryonic and post-embryonic growth in tomato [21]. Moreover, it was shown to mediate seed development by affecting the onset of endosperm cellularization [22]. A previous study revealed that, akin to its homolog *AtRan3*, longan Ran might have similar roles in cell proliferation during somatic embryogenesis [12]. Notably, *DlRan3A* and *DlRan3B* indicated functional similarity and specificity in transcriptional regulation during longan somatic embryogenesis [14,15]. Here, based on expression profiling of *DlRan3A* and *DlRan3B* at different development stages of seeds, fruits, and other tissues, we found that both genes were highly expressed in seeds and pulp (aril), as well as in the young embryos of early-stage zygotic embryo development. Additionally, they may positively regulate fruit pulp swelling. Importantly, based on the previous similar findings of their roles in late embryogenesis (cotyledon embryos), *DlRan3A* and *DlRan3B* might regulate early embryo development, especially the formation of cotyledons.

Proteins involved in cell wall synthesis and expansion in plants are closely related to plant development including embryo and fruit development [23]. For instance, XET/XTH, an important enzyme for cell wall loosening, has a key role in plant somatic embryogenesis [24]. Additionally, the genes encoding for cellulose synthase-like protein AtCSLA7, hydroxyproline-rich glycoprotein RSH (root-shoot-hypocotyl-defective), and AtEXT3 (extensin 3), are essential for cell wall structure and normal embryo development in Arabidopsis [25,26]. The current study suggests that longan *Ran* expression led to up-regulation of numerous cell wall biosynthesis and expansin/extensin genes, indicating the role of *Ran* in cell wall development. Additionally, during the fruit expansion stage, the cell wall needs to turn loose. We found that *DlRan3A* and *DlRan3B* were highly expressed in longan pulp, and their expression levels increased with the expansion of longan pulp (aril), along with several up-regulated genes related to cell wall loosening or fruit ripening (encoding expansins, XETs, XTHs, etc.). This indicates the potential role of *Ran* in fruit development by enhancing the accumulation of cell wall-loosening proteins. The current study provides significant new insights for *Ran* function during embryo and pulp (aril) development. However, the potential mechanism(s) of *Ran*-mediated transcript accumulation of cell wall-related genes during early embryo and pulp (aril) development needs to be further revealed.

### 3.2. pDlRan3A Driven Expression of DlRan3A Led to Stunted Plant Growth, Higher Root Hair Density, Abnormal Fruits, and Bigger Seeds, Potentially via Partial Regulation of Expansin- and Extensin-like Genes

Ran GTPase is closely related to both vegetative and reproductive growth in plants. Expression of wheat *TaRAN1* prolongs the life cycle, elevates mitotic index, decreases lateral roots, increases primordial tissue, and enhances auxin hypersensitivity [6]. Similarly, ectopic expression of *FaRan* in Arabidopsis increases axillary buds and reduces apical dominance, suggesting its potential role in the initiation of meristem and subsequent growth and development [19]. In this study, p*DlRan3A*-controlled expression of *DlRan3A* led to stunted plant growth, increased root hair density, abnormal fruits, and larger seeds, suggesting the important role of *Ran* in certain organs. It seems that an excessive or ectopic presence of *Ran*, beyond its optimal concentration, may significantly affect plant development.

The agronomic characteristic dwarfism, such as reduced height and thick stems, is often desirable to bolster lodging resistance [27]. The current results suggest that p*DlRan3A*-driven expression of *DlRan3A* can be an additional functional factor for stunted plant growth. Importantly, stunted or dwarfed plant growth has also been linked to transcriptional regulation of genes related to cell wall synthesis and expansion [28], which is consistent with our finding that two *expansin* genes, *AtEXLA2* Niben101Scf03913g01045 and *OsEXPA4* Niben101Scf20887g00008, were, respectively, up-regulated and down-regulated in the PA_A/P35S_A comparison. Despite the fact that the suppressed expression of the *expansin* gene is known to reduce plant growth while the overexpression does not necessarily promote it [28,29,30], our finding highlights the role of *DlRan3A* in the regulation of stunted plant growth and provides a novel clue for understanding the molecular mechanism of *Ran* in the activation or suppression of *expansin* genes.

Root hairs, offering the plant a competitive advantage in the acquisition of water and nutrients from the soil, can enhance plant tolerance to abiotic stress [31]. Though previous studies have revealed the functional involvement of small GTPases in root hair growth [32,33], detailed investigations are required to understand the underlying mechanism(s). In particular, the role of Ran GTPases has not yet been reported in root hair growth. Here, we found that three extensin-like genes were up-regulated in PA_A (compared to P35S_A), and down-regulated in PB_B (compared to P35S_B), while both types were significantly enriched in pathways of cell wall organization or biogenesis. Based on the phenotypes of transgenic plants, we propose that *DlRan3A* and *DlRan3B*, driven by their native promoters, might play different roles in cell wall organization or biogenesis in longan tissues, especially via regulation of extensin-like genes. Emerging evidence suggests that extensin- and extensin-like proteins are essential for cell wall self-assembly and, hence, root hair development, such as in tomato [34], barley [35], Arabidopsis [36], etc. Here, we showed that *DlRan3A* plays a very specific role in root hair development via up-regulation of extensin-like genes, but further studies are required to discover and validate the downstream effectors of *Ran* in root hair development.

In addition, one of the three extensin-like genes was annotated as pistil-specific extensin-like protein gene ‘*PELP*’ (Niben101Scf03036g00006). Notably, extensins have been reported in reproductive development in some plants. For instance, the class III pistil-specific extensin-like proteins (PELPIIIs) of Nicotiana were associated with interspecific incompatibility, specific inhibition of pollen tube, and growth [37,38]. p*DlRan3A*-driven expression of *DlRan3A* led to abnormal fruits, larger seeds, a reduced seed setting rate, and increased seed weight, coinciding with increased and abnormal expression of *PELP* in PA_A. Thus, *DlRan3A* and *PELP* might be closely interrelated and involved in reproductive development, providing new perspectives on *Ran*-mediated transcriptional regulation of plant reproductive processes.

### 3.3. DlRan3A and DlRan3B Function in Stress Tolerance by Regulating Different Stress-Responsive Genes

A close association between *Ran* and plant responses to environmental stresses, such as salinity [39], osmotic stress [8], drought [7], low temperatures [9,40], aluminum toxicity [41], and oxidative stress [11], has been elucidated in numerous studies. However, the understanding of the underlying mechanisms is slowly improving, and there have been rare reports about the role of longan *Ran* in defense responses. In this study, using transcriptome analysis, we revealed numerous DEGs related to cell wall biosynthesis and stress response (*PER*, *aquaporin*, *AS*, *GST*, *HSP*, *DNAJ*, etc.), as well as stress-related transcription factor (MYB, NAC, WRKY, ERF, GRAS, bHLH, etc.) DEGs. The data strongly suggest the important roles of *Ran* in a complex stress regulatory network. We found that the tobacco overexpressing *DlRan3A* (P35S_A and PA_A) were more tolerant to abiotic stress (salinity, osmotic, and heat stress). Conversely, tobacco overexpressing *DlRan3B* (P35S_B and PB_B) exhibited relatively poor tolerance compared to the P35S_A and PA_A lines. In particular, compared to WT and *DlRan3B* overexpressing tobaccos, P35S_A tobaccos showed a little more sensitivity to ABA, which plays a critical role in various stress responses. More studies are needed to further elucidate the role of *DlRan3A* in relationship with ABA accumulation and stress (salinity, osmotic, and heat stress) tolerance. 

Previous studies in rice suggesting that *RAN* overexpression improved cold tolerance are partially supported by our results. Despite *Ran’s* associations with cell division, the hormone BR might also participate in *Ran*-regulated cold tolerance. Notably, many previous studies have shown BR-induced cold tolerance in plants, such as in Arabidopsis and *Brassica napus* [42], *Cucumis sativus* [43], *Chorispora bungeana* [44], *Elymus nutans* [45], *Medicago truncatula* [46], etc. Importantly, plants overexpressing longan *Ran* showed increased accumulation of *EXORDIUM* transcripts than the WT (Figure S7), and well-characterized BR-responsive genes, such as *expansins*, *XETs*, *aquaporins*, and *ASs* [47,48,49], were significantly up-regulated in plants with increased longan *Ran* expression. The phenotypic observations, together with the transcriptome analysis of the BR-responsive genes, suggest a possible role of longan *Ran* in activated BR signaling and BR-mediated plant defense mechanisms, especially in cold tolerance. 

Despite the similarities, the functional divergence between the two members of the longan *Ran* family in stress responses (salinity, osmotic, and heat) might be partially attributed to the inhibition of stress-regulated transcription factor (WRKY, ERF, GRAS, bHLH, C3H, C2H2, etc.) genes in P35S_B or PB_B. Moreover, the activation of oxidative stress-related genes (*PER*, *GST*, *GPX*, *PPO*, *RBOH*, etc.) can stimulate the antioxidant defense system in P35S_vA and PA_A, resulting in the differences between *DlRan3A* overexpressing tobaccos and *DlRan3B* overexpressing ones in terms of their stress responses (Figure S13). Plant RBOHs are the key enzymes that catalyze the generation of reactive oxygen species (ROS) during plant defense responses and are involved in the modulation of root growth [50]. Plant CBL (calcineurin B-like) and CIPK (CBL-interacting protein kinase) proteins, form one of the important Ca^2+^ decoding complexes to decipher Ca^2+^ signals elicited by environmental challenges [51]. Rac/Rop GTPases and CBL-CIPKs involve integration of calcium signaling into ROS regulation via direct interaction with RBOH [52]. Using GO analysis, we showed that *CIPK* was significantly down-regulated in PA_A, while the RBOH was up-regulated. Considering the fact that RBOH acts as a convergence point targeted by a complex regulatory network, these data endorse the notion that the resistant phenotypes and associated molecular changes in plants overexpressing *DlRan3A* are at least partly due to the Ran–RBOH–CIPK regulatory network. This new evidence suggests the involvement of *Ran* in plant defense responses via calcium signaling and ROS regulation.

## 4. Materials and Methods

### 4.1. Plant Materials

The different tissues of longan were collected from Fujian Agriculture and Forestry University in Fuzhou. All materials were mixed samples from at least six rootstock longan plants, collected and stored at −80 °C for further studies. *Nicotiana benthamiana* tobaccos were used for stable genetic transformation.

### 4.2. Gene Expression Analysis

Total RNA was extracted using the RNAprep Pure Plant Kit (TIANGEN Code, DP441, Beijing, China) or total plant RNA extraction kit (BioTeke Code, RP3312, Beijing, China) following the manufacturer’s protocol. cDNAs were synthesized using the PrimeScript^TM^ Perfect Real-Time RT Reagent Kit (TaKaRa Code, RR037A (Dalian, China)). Quantitative real-time PCR analysis (qPCR) was performed to evaluate the transcript levels of the *DlRan3A* and *DlRan3B* genes in longan tissues, during zygotic embryo and pulp developments. Typical reactions were prepared using the SYBR Premix Ex Taq kit (Takara) and all the qPCR reactions were performed in triplicate. QPCR assays were implemented using the LightCycler 480 qPCR instrument (Roche Applied Science, Basel, Switzerland) and cycling conditions were chosen according to the manufacturer’s protocol. The expression profiles of *DlRan3A* and *DlRan3B* in longan tissues were quantified using three pre-microRNAs (*pre-miR167f3p*, *pre-miR171f*, and *pre-miR394a*) as the reference genes; and the expression levels during zygotic embryo development were quantified using the 2^−ΔΔCT^ method, with longan *Fe-SOD* as a reference gene. The expression levels of *DlRan3A* and *DlRan3B* in other longan samples were quantified using the internal standards as described previously [53]. Primer names and sequences are provided in Table S1.

### 4.3. Vector Construction

The coding sequences of *DlRan3A* (JQ775539) and *DlRan3B* (JQ279697) were PCR-amplified from longan cDNA using primers with *BamH* I/*Sal* I restriction sites at the 5′/3′ ends, respectively. To generate expression constructs pCAMBIA1301-*35S*-*DlRan3A* (hereinafter, referred to as ‘35S_A’) and pCAMBIA1301-*35S*-*DlRan3B* (hereinafter, referred to as ‘35S_B’), the PCR-amplified products were in-fusion cloned into the corresponding sites of pCAMBIA1301SN vector (modified by Feng [54]) having a *35S* promoter and nos terminator, respectively. p*DlRan3A* and p*DlRan3B* were amplified from longan DNA, using primers with *EcoR* I*/Kpn* I restriction enzyme sites at the 5′/3′ ends. The *35S* promoter was removed from the construct 35S_A and 35S_B by *EcoR* I and *Kpn* I digestion, and the amplified products were in-fusion cloned into the corresponding sites of *35S*-removed vectors, respectively, to generate expression constructs pCAMBIA1301-p*DlRan3A(1256bp)*-*DlRan3A* (hereinafter, referred to as ‘PA_A’) and pCAMBIA1301-p*DlRan3B (1569bp)*-*DlRan3B* (hereinafter, referred to as ‘PB_B’). To construct pCAMBIA1301-p*DlRan3A(1256bp)*-*GUS* (hereinafter, referred to as ‘PA_GUS’) and pCAMBIA1301-p*DlRan3A(1256bp)*-*GUS* (hereinafter, referred to as ‘PB_GUS’), p*DlRan3A* and p*DlRan3B* were amplified using primers with *Hind* III*/Nco* I restriction sites, and then in-fusion cloned into pCAMBIA1301 with removed *35S*. These expression constructs were transformed into *N. benthamiana* using *Agrobacterium* strain EHA105. Primer sequences for the isolation of the *DlRan3A* and *DlRan3B* genes, as well as their promoter sequences, are provided in Supplementary data Table S2. The design of these constructs is depicted in Supplementary data Figure S1. 

### 4.4. Generation of T2 Transgenic Tobacco Plants

The *Agrobacterium*-mediated transformation of tobacco leaf segments and regeneration of T0 transgenic tobaccos were performed following the method by Feng [54]. To generate T1 tobacco plants, the T0 lines of P35S_A, PA_A, P35S_B, and PB_B plants were grown in a growth chamber until flowering, and then the self-pollination stage. The resulting generations were selected against hygromycin pressure (40 mg/L), PCR verified, and validated with GUS staining by using GUS histochemical assays kit (Real-Times, Beijing, China). Similarly, the final T2 lines, used for further analysis, were obtained through self-pollination of T1 plants and then subjected to similar screening. The single T2 line of P35S_A or PA_A was obtained from T1 lines exhibiting the highest expression levels of *DlRan3A*. Similarly, the single T2 line of P35S_B or PB_B was obtained from T1 lines exhibiting the highest expression levels of *DlRan3B* (Figure S2). The used primer sequences are provided in Table S3.

### 4.5. Phenotypic Analysis

The effects of *DlRan3A* or *DlRan3B* overexpression were analyzed by scoring a range of specific plant phenotypes: flowering time, plant height, blade generation rate, and the development of roots, flowers, fruits, and seeds. The data for phenotype analysis were acquired from a minimum of fifteen independent plants. The tobacco root tips were stained by propidium iodide as described previously and observed by laser scanning confocal microscopy (Olympus, Tokyo, Japan; FV1200) [40].

### 4.6. Analysis of Environmental Stress Response

Seeds of T1 lines of P35S_A, PA_A, P35S_B, and PB_B tobacco plants (T2 lines) were germinated on MS medium with 100 mM NaCl, 200 mM mannitol, or 10 μM ABA. All seedlings were cultivated for 10 d in a chamber of 25 °C. For the heat and cold treatments, the tobacco seeds germinated on MS medium were exposed to 35 or 15 °C, respectively, for 6 h and then cultivated in the same condition as mentioned above. The control transgenic and non-transgenic tobaccos groups were cultivated without any treatments.

### 4.7. RNA-Sequencing

A cDNA library was generated from a pool of equal quantities of total RNA from 21 d cultivated tobacco seedlings of non-transgenic WT and transgenic T2 lines, 35S_A, PA_A, P35S_B, and PB_B, respectively. Plant samples were sent to the Novogene company (Beijing, China) for RNA extraction, cDNA library preparation, sequencing, quality control, reads mapping to the reference genome, and the gene expression quantification. RNA-seq of the mRNA and libraries was performed on an Illumina^®^ HiSeq™ 2000 platform according to Cui’s method [55]. DEGs were screened according to their expression profiles meeting the following criteria: |log2| (transgenic lines/corresponding control) > 1 and the adjusted *p* value < 0.005. Expression profiles of the screened DEGs were visualized by heatmap using the TBtools software (v0.6673) [56]. The iTAK database was used to define the transcription factor family according to relative rules [57,58]. Hormone-related genes and stress-related genes were analyzed based on annotations in the TAIR (The Arabidopsis Information Resource) database. The GO and KEGG enrichment analyses of the DEGs were conducted following the method by Zhao [59]. The GO terms and KEGG pathways with corrected *p*-values < 0.05 were considered significantly enriched.

### 4.8. qPCR Validation

Total RNAs were isolated from transgenic tobaccos using the TriPure Isolation Reagent (Roche) following the manufacturer’s protocol. cDNA synthesis and qPCR analysis were conducted as above described, using the 2^−ΔΔCT^ method with *NbEF1a* as a reference gene [16]. Primer sequences are provided in Table S4.

## 5. Conclusions

In this study, we investigated the expression and function of longan Ran GTPase genes *DlRan3A* and *DlRan3B.* The results of the study indicated that *DlRan3A* and *DlRan3B* have important roles in longan early embryo and pulp development. Heterologous expression of *DlRan3A* and *DlRan3B* by employing *35S* or *Ran* promoters, combined with *Ran*-overexpression transcriptome of transgenic plants, showed that *DlRan3A* and *DlRan3B* regulate cell wall-related genes to affect plant growth. Particularly, p*DlRan3A*-driven expression of *DlRan3A* led to stunted plant growth, higher root hair density, abnormal fruits, and bigger seeds, potentially via partial regulation of the expansin- and extensin-like genes. Furthermore, *DlRan3A* and *DlRan3B* might function via shared molecular mechanisms in cold stress response but differed in conferring plants with salinity, osmotic, and heat stress tolerance or sensitivity, by up-regulated or down-regulated different stress- or hormone-related genes. Overall, the present study provided a widespread characterization on longan Ran GTPase genes *DlRan3A* and *DlRan3B*, highlighting the associated gene expression mechanisms incorporated by *Ran*.

## Data Availability

The RNA sequencing data have been uploaded to the NCBI (National Center for Biotechnology Information) SRA (Sequence Read Archive) under accession number PRJNA607013.

## Supplementary Materials

The following supporting information can be downloaded at: https://www.mdpi.com/article/10.3390/plants13040480/s1, Figure S1: Constructed vectors of *DlRan3A* and *DlRan3B*; Figure S2: Gene-relative expression in transgenic modified tobacco (T1 lines); Figure S3: Height (a) and leaf number (b) of transgenic tobacco; Figure S4: Phenotype of transgenic tobacco root (7 d and 14 d); Figure S5: Hundred-grain weight of transgenic tobacco; Figure S6: GUS staining of transgenic tobacco seedlings (T1 lines); Figure S7: GUS staining of flowers, fruits, and seeds of transgenic tobacco; Figure S8: Expression profiles of the shared DEGs in transgenic lines; Figure S9: Expression profiles of DEGs classified as genes encoding transcription factors (TFs) (a) and hormone-related genes (HRGs) (b); Figure S10: Expression profiles of DEGs involved in cell wall development; Figure S11: Expression profiles of stress-related DEGs; Figure S12: Mapman software (v3.6.0) visualization of stress-related genes; Figure S13: Expression profiles of the antioxidant-defense-related DEGs; Table S1: Sequences of primers used for qPCR analysis of *DlRan3A* and *DlRan3B* in longan; Table S2: Primer sequences used for *DlRan3A* and *DlRan3B* constructs for genetic transformation of *Nicotiana benthamiana*; Table S3: Sequences of primers used for qPCR detection in transgenic tobaccos; Table S4: Primer sequences used for qPCR verification; Table S5: Comparison of reads by transcriptome sequencing to reference genome; Table S6: Differentially expressed genes (DEGs) of the GM *N. benthamiana* lines; Table S7: Shared DEGs between the P35S_A and P35S_B lines; Table S8: Shared DEGs between the P35S_A and PA_A lines; Table S9: Shared DEGs between the P35S_B and PB_B lines; Table S10: Significant GO enrichment pathways of the genetically modified (GM) *N. benthamiana* lines; Table S11: Significant KEGG enrichment pathways of the GM *N. benthamiana* lines.
