# Supplementary material for: Functional and Transcriptome Analysis Reveal Specific Roles of Dimocarpus longan DlRan3A and DlRan3B in Root Hair Development, Reproductive Growth, and Stress Tolerance
Source: Plants (Basel). 2024 Feb 7;13(4):480. doi: 10.3390/plants13040480 (PMC10891736; doi:10.3390/plants13040480)
Supplement: Supplementary file 1 [file plants-13-00480-s001.zip › plants-2825733-supplementary.pdf]

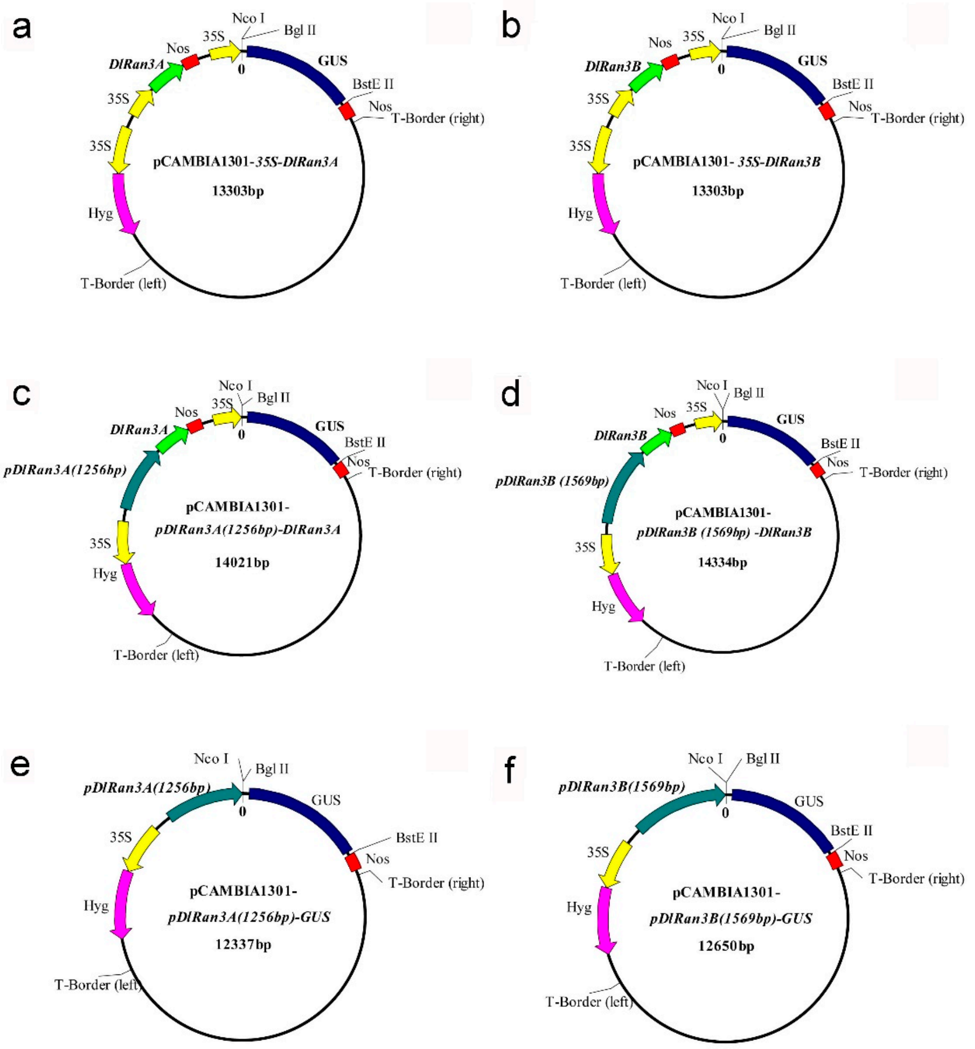


**Figure S1. Constructed vectors of *DlRan3A* and *DlRan3B*.** **a** pCAMBIA1301 - 35S - DlRan3A (P35S_A). **b** pCAMBIA1301 - 35S - DlRan3B (P35S_B). **c** pCAMBIA1301 - pDlRan3A (1256bp)- DlRan3A (PA_A). **d** pCAMBIA1301 - pDlRan3B (1569bp) - DlRan3B (PB_B). **e** pDlRan3A (1256bp) – GUS (PA_GUS). **f** pCAMBIA1301 - pDlRan3B (1569bp) – GUS (PB_GUS).

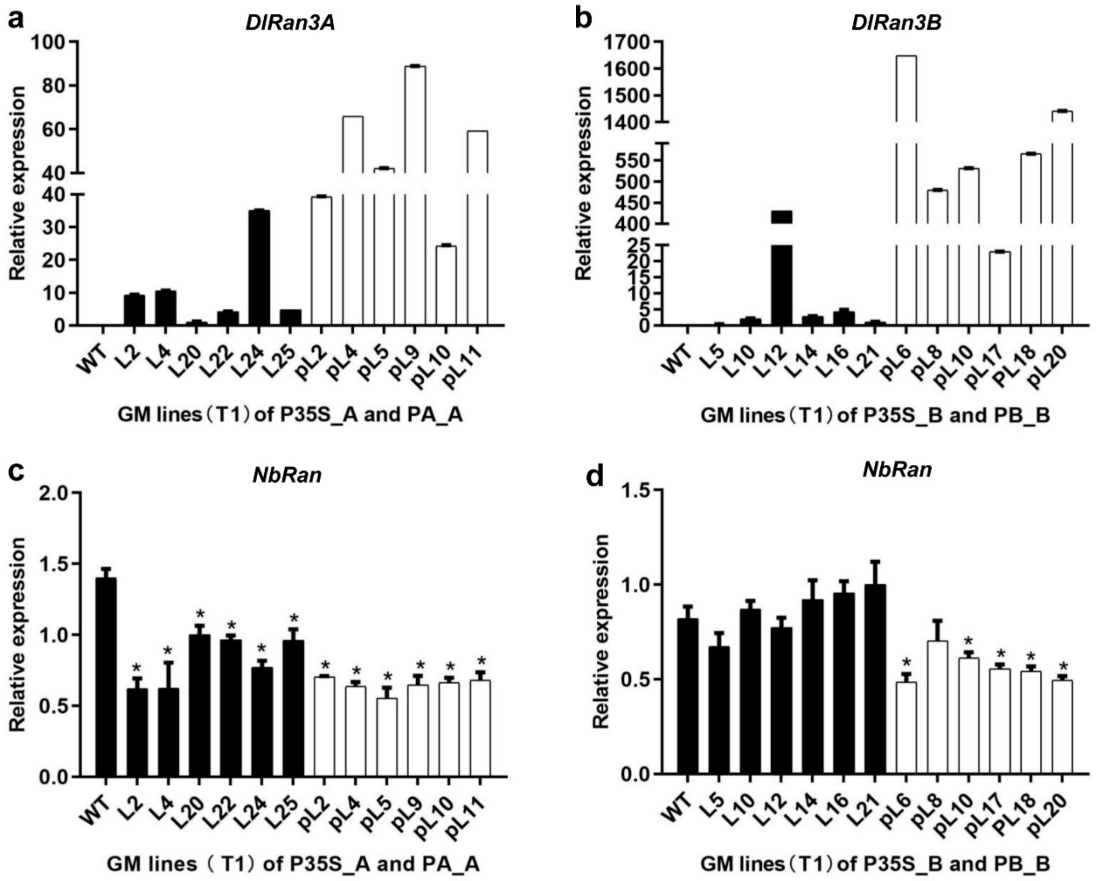


**Figure S2. Gene relative expression in transgenic modified tobacco (T1 lines).** a Relative expression of *DlRan3A* in P35S_A and PA_A T1lines; b Relative expression of *DlRan3B* in P35S_B and PB_B T1lines; c Relative expression of *NbRan* in P35S_A and PA_A T1lines; d Relative expression of *NbRan* in P35S_B and PB_B T1lines; significant differences in contrast to the control (WT) are indicated with an asterisk (*) and were assessed by a one-way ANOVA test (* $p < 0.05$)

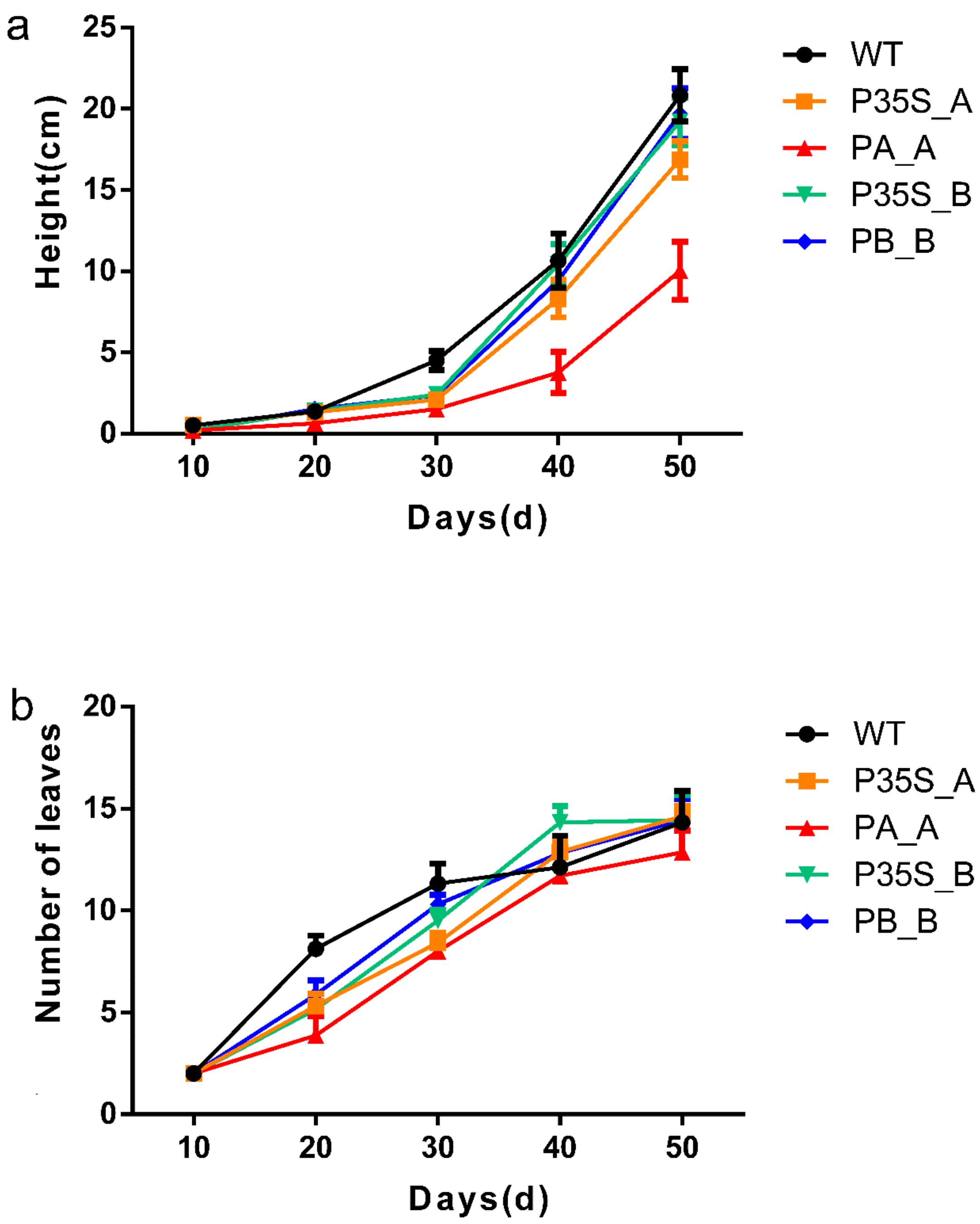


**Figure S3. Height (a) and leaf number (b) of transgenic tobacco.**

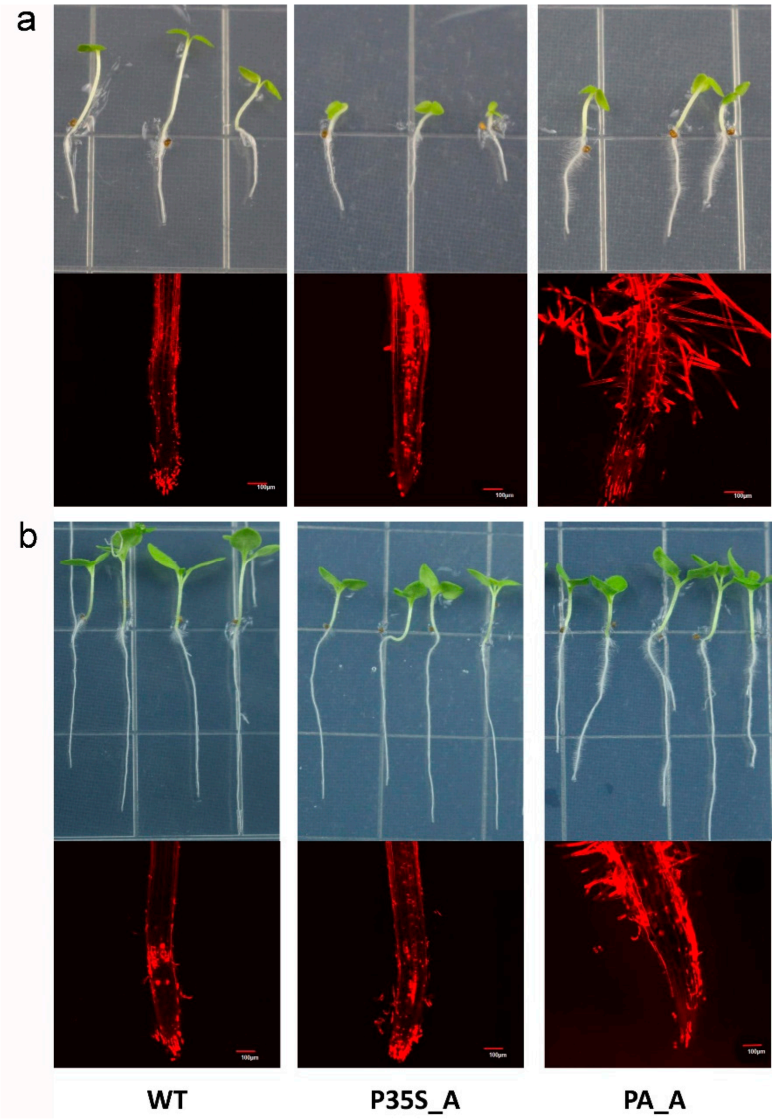


**Figure S4. Phenotype of transgenic tobacco root (7d and14d).** Transgenic tobaccos were germinated and vertically cultivated for 7d (**a**) and 14d (**b**). WT and T2 lines of P35S_A and PA_A were displayed in the graph from left to right in turn. The upper images illustrated transgenic tobaccos germinated and vertically cultivated on MS medium for 7d and 14d, respectively, and the lower images illustrated the 7 day- and 14 day-cultivated tobacco roots stained with propidium iodide (bar =100 μm), respectively.

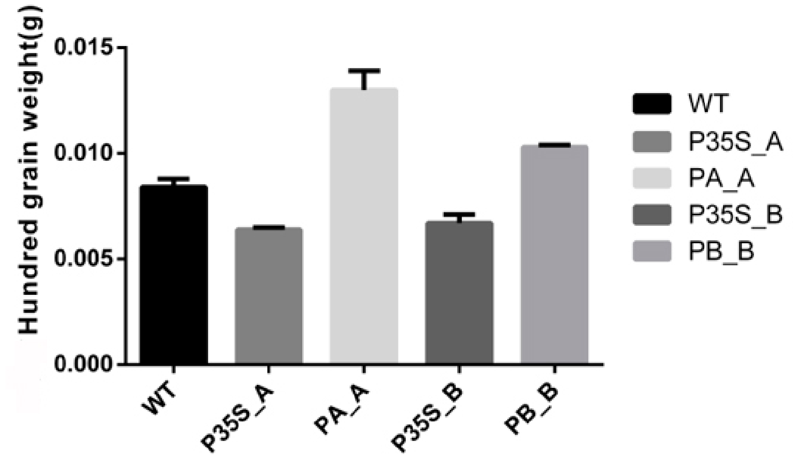


**Figure S5. Hundred grain weight of transgenic tobacco.**

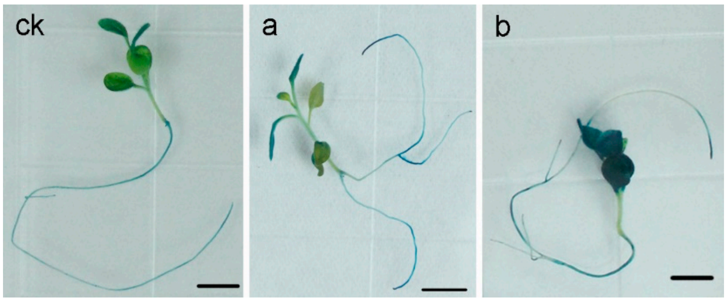


**Figure S6. GUS staining of transgenic tobacco seedlings (T1 lines). ck**: 1301. **a**: PA-GUS. **b**: PB-GUS. Bar = 5 mm.

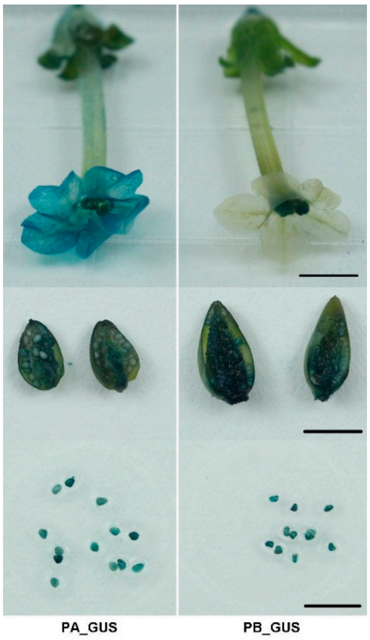


**Figure S7. GUS staining of flowers, fruits and seeds of transgenic tobacco (T0 lines).** Bar = 5 mm.

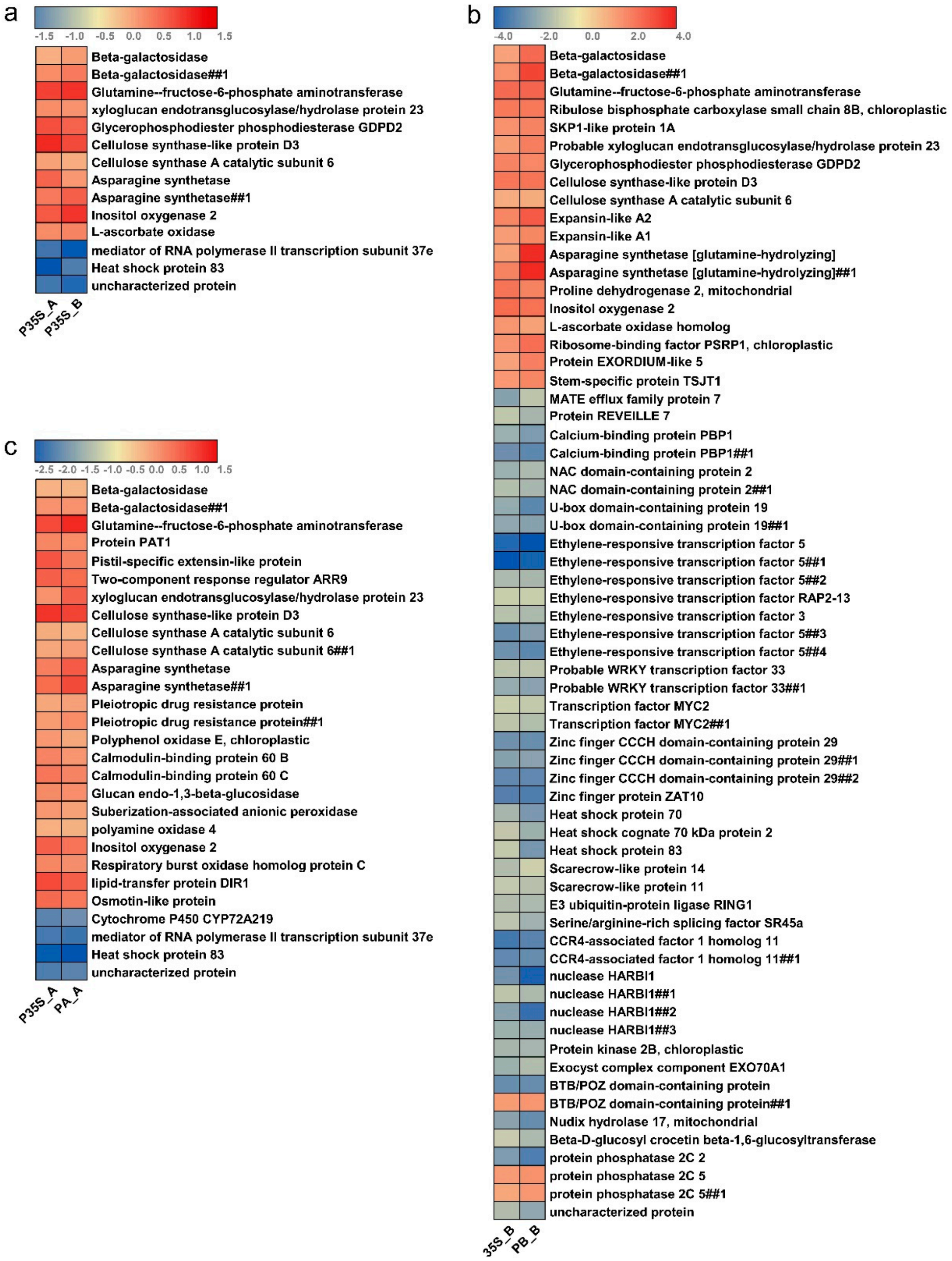


**Figure S8. Expression profiles of the shared DEGs in transgenic lines.**

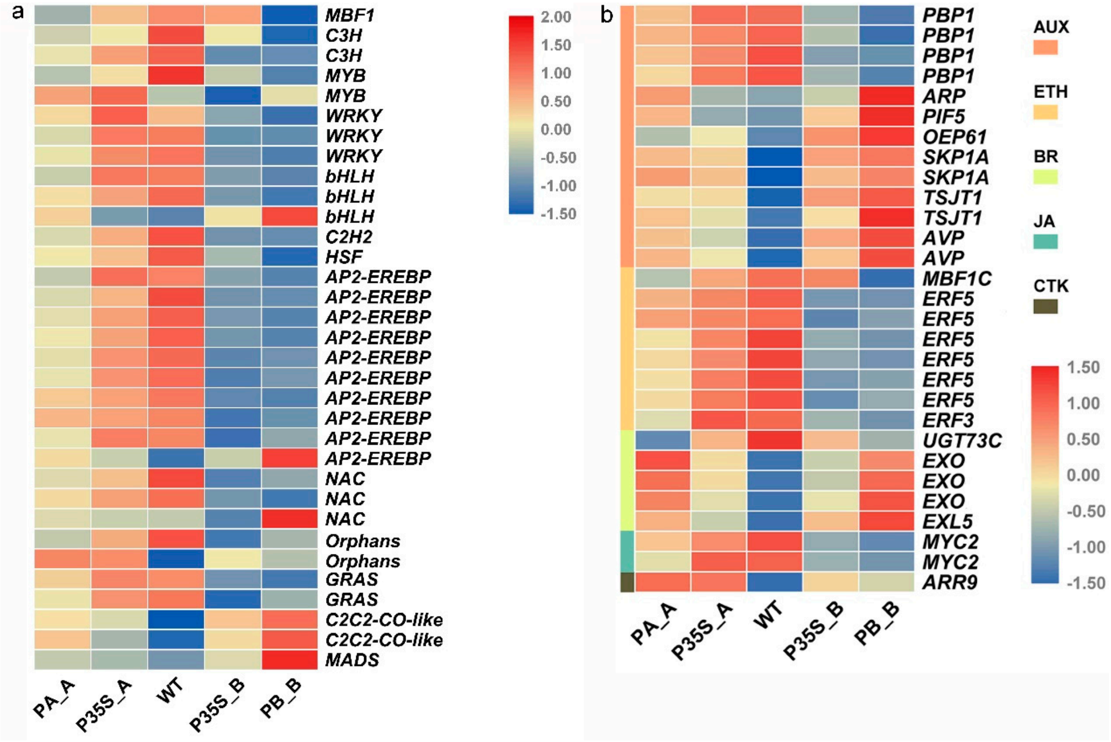


**Figure S9.** Expression profiles of the DEGs classified as genes encoding transcription factors (TFs) (a) and hormone-related genes (HRGs) (b).

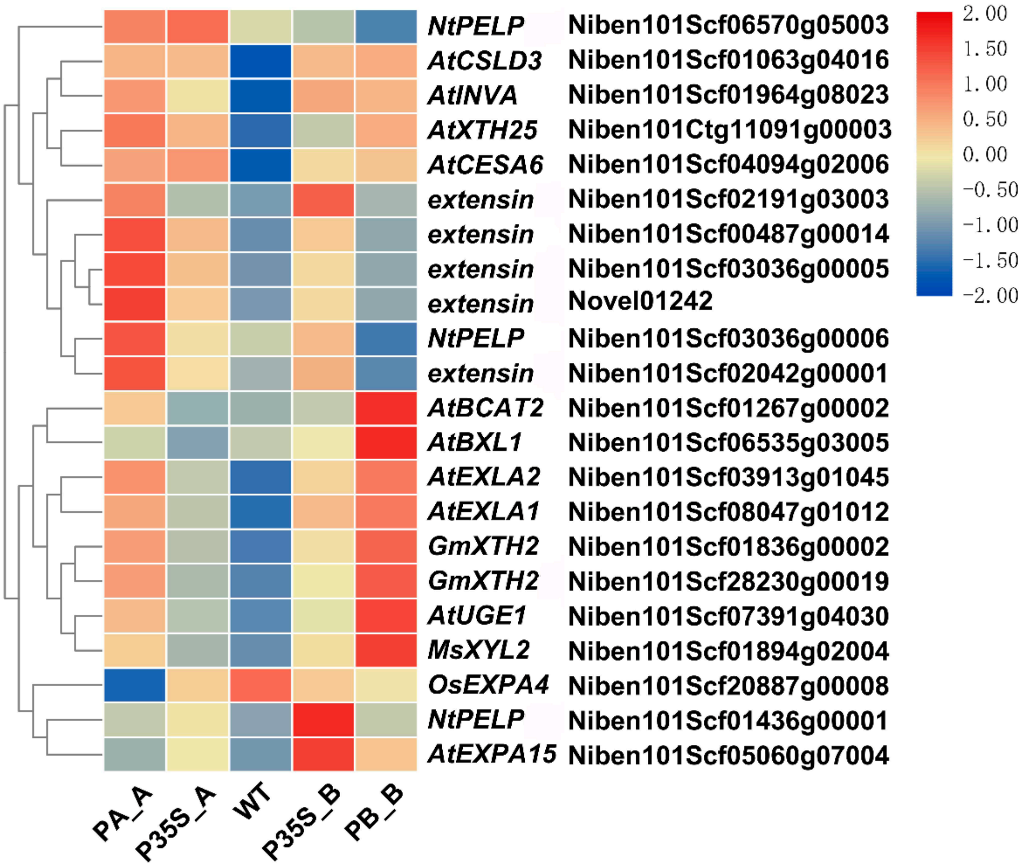


**Figure S10.** Expression profiles of DEGs involved in cell wall development.

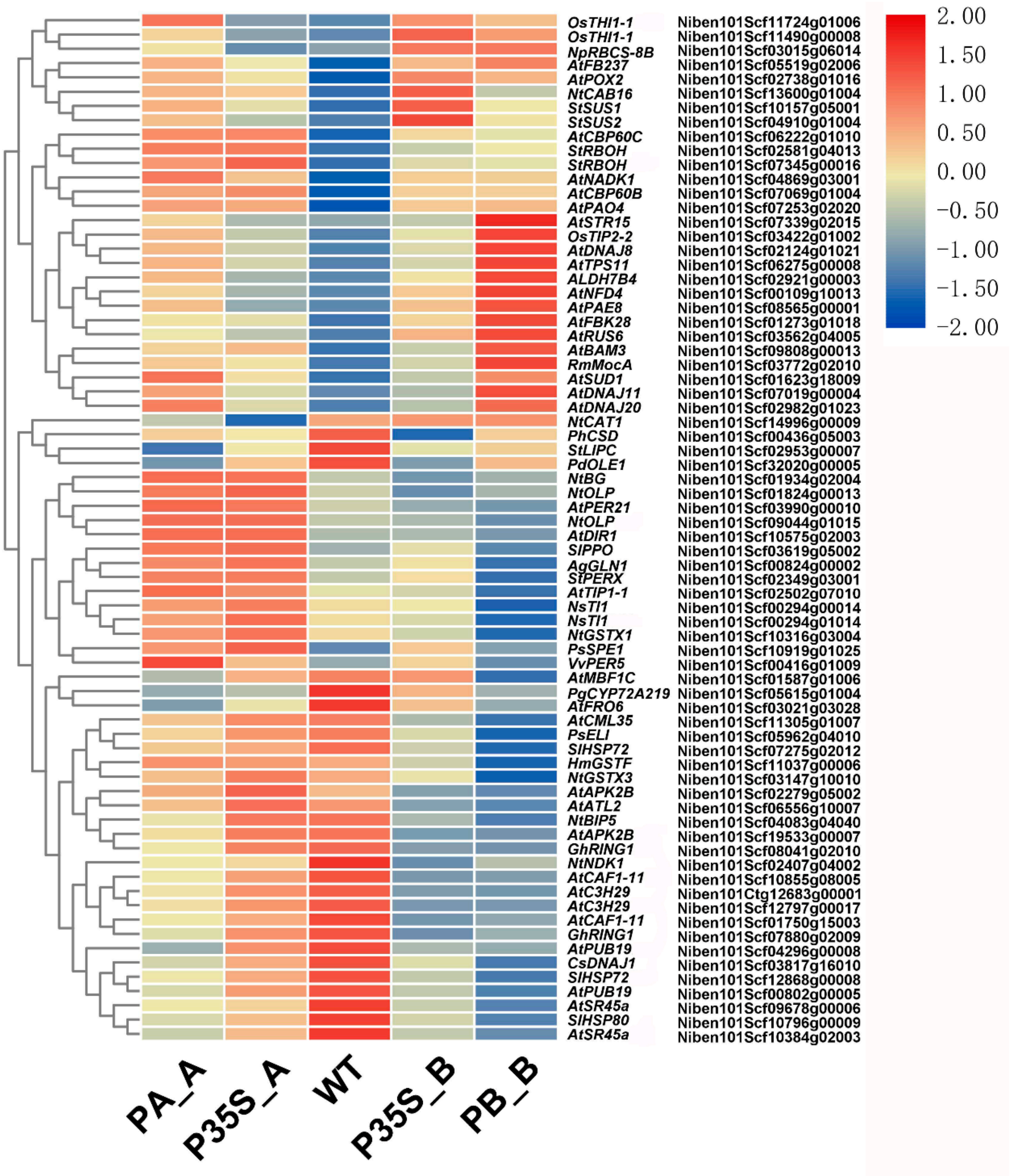


**Figure S11. Expression profiles of stress-related DEGs.**

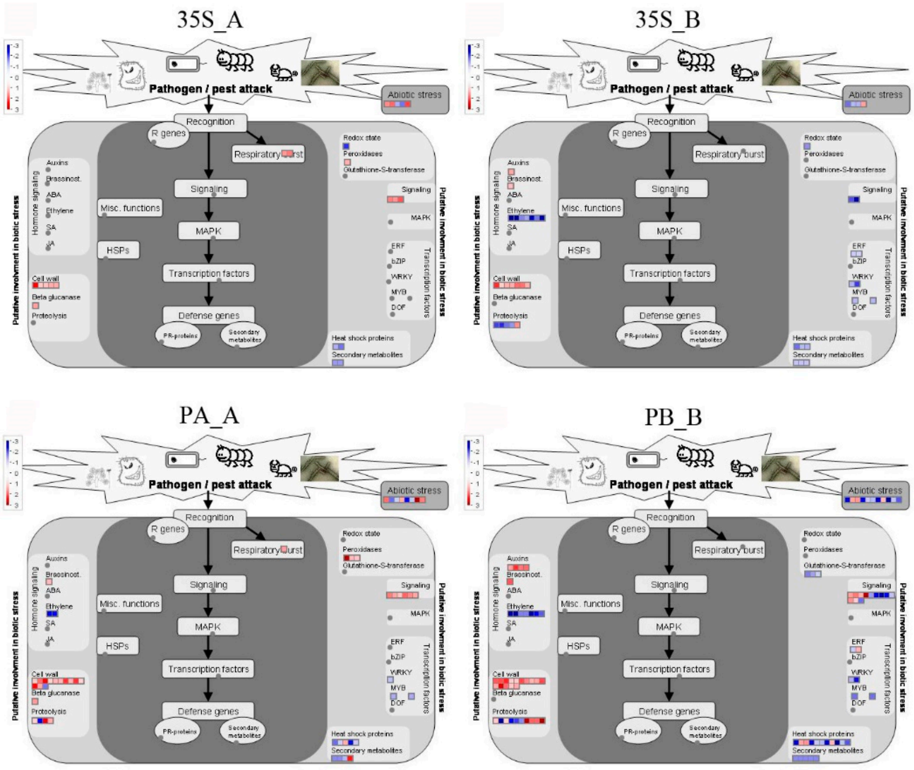


**Figure S12. Mapman software visualization of stress-related genes.** Genes differentially expressed (|Log2(FC)| ≥ 1 and Padj ≤ 0.005) of transgenic tobacco plants overexpressing longan Ran were represented according to their respective Log2 rescaled fold change. Red color represents up-regulated genes and blue color represents down-regulated genes. The dark gray rectangle includes genes directly involved in biotic stress responses and the light gray rectangle includes genes potentially involved in biotic stress responses. The medium gray rectangle includes genes involved in abiotic stress responses. ABA, abscisic acid; bZIP, basic region leucine zipper; DOF, DNA-binding with one finger; ERF, ethylene responsive factor; HSP, heat shock protein; JA, jasmonic acid; MAPK, mitogen activated protein kinase; MYB, v-myb avian myeloblastosis viral oncogene homolog; PR, pathogenesis-related; R, resistance; SA, salicylic acid.

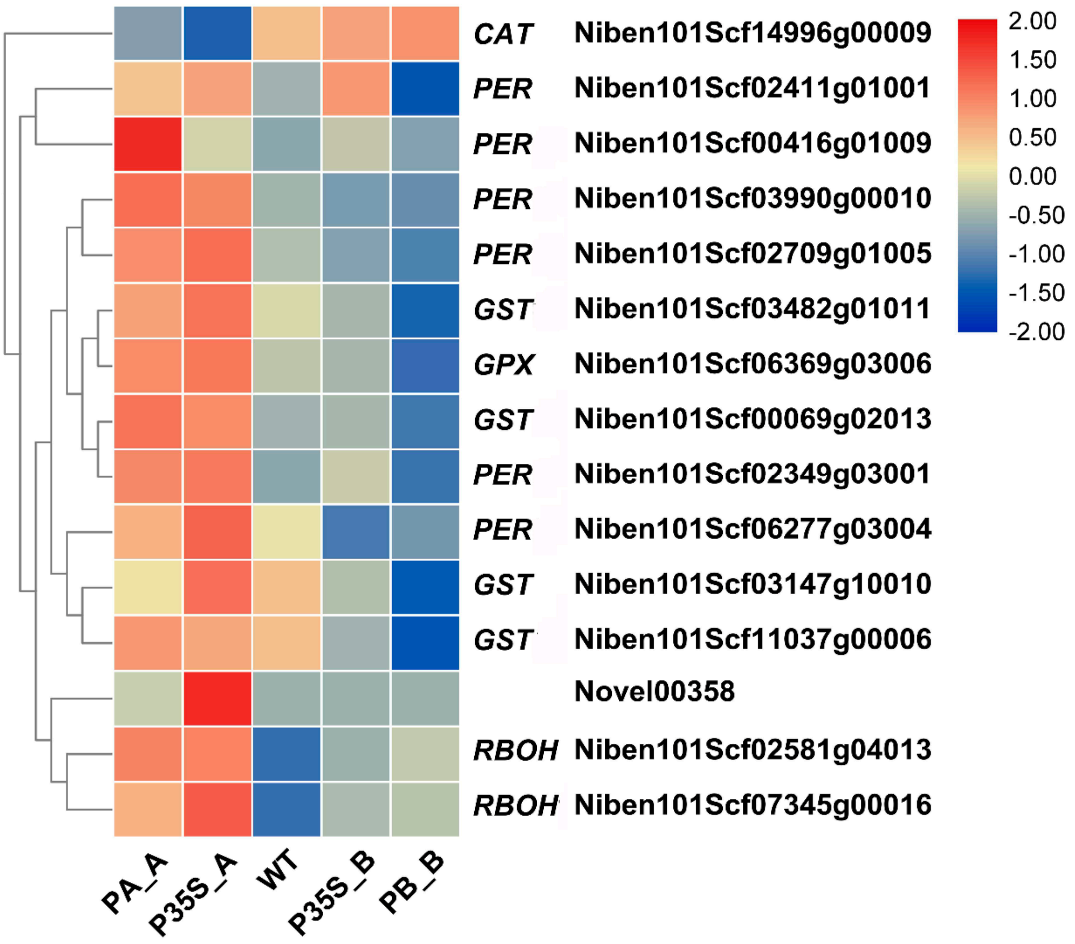


**Figure S13. Expression profiles of the antioxidant-defense-related DEGs.**

**Table S1. Sequences of primers used for qPCR analysis of *DlRan3A* and *DlRan3B* in longan.**

| Primer Name | Primer Sequence (5′→3′) |
|---|---|
| DlRan3A-QF | GCATGAGGCTGAACTGCAA |
| DlRan3A-QR | TCCCCATCCTGCTGTTTTAC |
| DlRan3B-QF | CATCATGAAGCTGAGCTTGC |
| DlRan3B-QR | CCAGCCTGCAACTGTTCTC |
| pre-miR167f3p (scaffold692)QF | TGAAGCTGCCAGCATGAT |
| pre-miR167f3p (scaffold692)QR | CCATCAATAGGTGAAACTGCC |
| pre-miR171f (scaffold760)QF | CGGTTCGGTTCAATGAGAA |
| pre-miR171f (scaffold760)QR | GATATCGGCACGGCTCAA |
| pre-miR394a (scaffold3884)QF | CGCATTCTGTCCACCTCC |
| pre-miR394a (scaffold3884)QR | AGAGAAGCCAACAGAGCTCAG |

**Table S2. Primers sequences used for *DlRan3A* and *DlRan3B* constructs for genetic transformation of *Nicotiana benthamiana*.**

| Primer name | Sequence (5'→3') |
|---|---|
| DlRan3A-(BamHI)F | TGGTACCCGGGGATCCATGGCTCTACCGAATCAGC |

| | |
|---|---|
| DlRan3A-(SalI)R | AACGCTGCAGGTCGACTTACTCGAATGCATCATCATC |
| DlRan3B-(BamHI)F | TGGTACCCGGGGATCCATGGCTTTGCCAAATCAGC |
| DlRan3B-(SalI)R | AACGCTGCAGGTCGACCTATTCAAATGTGTCATCATCGTC |
| pDlRan3A-(EcoRI)F | CCATGATTACGAATTCCGGAGAGTGTTGAAGGCC |
| pDlRan3A-(KpnI)R | CTAGAGGATCCCCGGGTACCTTCTTCTTGGTTTGGTTCTGAG |
| pDlRan3B-(EcoRI)F | CCATGATTACGAATTCAACAACTACCTACCAATGAGCG |
| pDlRan3B-(KpnI)R | CTAGAGGATCCCCGGGTACCGAGAGCTGTCCTTGAGAAAG C |
| pDlRan3A-(HindIII) F | GCAGGCATGCAAGCTTCGGAGAGTGTTGAAGGCC |
| pDlRan3A-(NcoI)R | CTCAGATCTACCATGGTTCTTCTTGGTTTGGTTCTG |
| pDlRan3B-(HindIII) F | GCAGGCATGCAAGCTTAACAACTACCTACCAATGAG |
| pDlRan3B-(NcoI)R | CTCAGATCTACCATGGGAGAGCTGTCCTTGAG |

**Table S3. Sequences of primers used for qPCR detection in transgenic tobaccos.**

| Primer name | Sequence (5'→3') | Function |
|---|---|---|
| DlRan3A-QF1 | GCAAGTGAAAGCCAAGCAA | *DlRan3A* primers used for qPCR detection of transgenic lines |
| DlRan3A-QR1 | TGACTAGCAGCAGCTTGCAGT | |
| DlRan3B-QF1 | CCTAGCTTCAAGCTCGTCATC | *DlRan3B* primers used for qPCR detection of transgenic lines |
| DlRan3B-QR1 | CCCAGCAGTAGAAACGGATT | |
| NbRanQF | GGCTTCTGAGGACTCTCTGTTC | Primers used for qPCR detection of endogenous *Ran* gene in transgenic lines |
| NbRanQR | CCAAATCGGTCCATCCAAAC | |

**Table S4. Primers sequences used for qPCR verification.**

| Primer name | Sequence (5'→3') | Gene ID |
|---|---|---|
| AtSKP1A-QF | CACTGACCCGCAAGCC | Niben101Scf02658g00012 |
| AtSKP1A-QR | TCTCGCCGTCGGAACTC | |
| VrAVP-QF | TGAGATGGCTGGAATGAGC | Niben101Scf05220g00005 |
| VrAVP-QR | AGAACCAATGGCAAATCCC | |
| AtCESA6-QF | CAAGGAAATGGGTCCCG | Niben101Scf04634g00004 |
| AtCESA6-QR | CACGGCGTTCCCTCAC | |
| NtPELP-QF | TGCCACATTTGAGGTGTTG | Niben101Scf03036g00006 |
| NtPELP-QR | GGTGGTGGAGAAGGAGAAGC | |
| SlPPO-QF | GGCAGAGTTCGCAGGAAG | Niben101Scf03619g05002 |
| SlPPO-QR | CGGGACCAGAGTCACCAC | |
| NtBG-QF | TTCTCCCAACAAGCAGCC | Niben101Scf01934g02004 |

| | | |
|---|---|---|
| NtBG-QR | CCGAACTGTCCCAAACTCC | |
| AtTIP1-1-QF | TTGGGCTGTTCGTGGC | Niben101Scf02502g07010 |
| AtTIP1-1-QR | TTACCGCCAACAAACGC | |
| StRBOHC-QF | TGTCAAAGCCACAGGGC | Niben101Scf02581g04013 |
| StRBOHC-QR | GCTGGCAAACCTCGGAG | |
| HmGSTF-QF | GCTTGGTCTAAGGCACTTGAG | Niben101Scf00069g02013 |
| HmGSTF-QR | CAACAATCACGCCTCGG | |
| NtGSTX1-QF | TTGCTGCTTCCGCTCG | Niben101Scf10316g03004 |
| NtGSTX1-QR | CTCTTGGGAGGCAACCC | |
| NtGPX4-QF | CCAGTTTGGTGGGCAGG | Niben101Scf06369g03006 |
| NtGPX4-QR | CAGCCTTGAAGCGAGTGC | |
| AtPER21-QF | TGGGACGAGTTCATTGTGTG | Niben101Scf03990g00010 |
| AtPER21-QR | GGTCAGGATTTGGTGTCGG | |
| AtPER10-QF | GCTCACACCATTGGATTTGC | Niben101Scf06277g03004 |
| AtPER10-QR | GTGTCGGGTCAGGATTTCC | |
| AtMYB59-QF | GGAGGAAATCAGAAGAGGTCC | Niben101Scf39382g00003 |
| AtMYB59-QR | GAGGAGTCATCTTGCCACG | |

**Table S5. Comparison of reads by transcriptome sequencing to reference genome.**

| Sample name | WT | P35S_A | PA_A | P35S_B | PB_B |
|---|---|---|---|---|---|
| Total reads | 42684446 | 55087298 | 45622008 | 52717818 | 50937938 |
| Total mapped | 38262505 (89.64%) | 49454712 (89.78%) | 41298764 (90.52%) | 47175126 (89.49%) | 45959763 (90.23%) |
| Multiple mapped | 2606302 (6.11%) | 3418317 (6.21%) | 2915443 (6.39%) | 3462580 (6.57%) | 3191092 (6.26%) |
| Uniquely mapped | 35656203 (83.53%) | 46036395 (83.57%) | 38383321 (84.13%) | 43712546 (82.92%) | 42768671 (83.96%) |
| Read-1 | 18417835 (43.15%) | 23749073 (43.11%) | 19830398 (43.47%) | 22584157 (42.84%) | 21989665 (43.17%) |
| Read-2 | 17238368 (40.39%) | 22287322 (40.46%) | 18552923 (40.67%) | 21128389 (40.08%) | 20779006 (40.79%) |

| | | | | | |
|---|---|---|---|---|---|
| Reads map to '+' | 17834532 (41.78%) | 23031003 (41.81%) | 19198815 (42.08%) | 21864927 (41.48%) | 21390603 (41.99%) |
| Reads map to '-' | 17821671 (41.75%) | 23005392 (41.76%) | 19184506 (42.05%) | 21847619 (41.44%) | 21378068 (41.97%) |
| Non-splice reads | 22857915 (53.55%) | 29452865 (53.47%) | 24625286 (53.98%) | 27987161 (53.09%) | 27375524 (53.74%) |
| Splice reads | 12798288 (29.98%) | 16583530 (30.1%) | 13758035 (30.16%) | 15725385 (29.83%) | 15393147 (30.22%) |

**Table S6. Differentially expressed genes (DEGs) of the GM *N. benthamiana* lines.**

| Comparison of lines | All DEGs | Up-regulated DEGs | Down-regulated DEGs |
|---|---|---|---|
| P35S_A vs WT | 56 | 46 | 10 |
| PA_A vs WT | 115 | 72 | 43 |
| P35S_B vs WT | 94 | 35 | 59 |
| PB_B vs WT | 186 | 107 | 79 |
| P35S_A vs P35S_B | 68 | 56 | 12 |
| PA_A vs P35S_A | 16 | 8 | 8 |
| PB_B vs P35S_B | 36 | 28 | 8 |
| PA_A vs PB_B | 82 | 59 | 23 |

**Table S7.** Shared DEGs between the P35S_A and P35S_B lines.

| Gene ID | Gene Expression | Annotation |
|---|---|---|
| Niben101Scf02363g00015 | UP | Beta-galactosidase |
| Niben101Scf10213g00009 | UP | Beta-galactosidase |
| Niben101Scf03253g02010 | UP | Glutamine--fructose-6-phosphate aminotransferase |
| Niben101Ctg13782g00003 | UP | Probable xyloglucan endotransglucosylase/hydrolase protein 23 |
| Niben101Scf00109g03008 | UP | Glycerophosphodiester phosphodiesterase GDPD2 |
| Niben101Scf03326g01009 | UP | Cellulose synthase-like protein D3 |

| | | |
|---|---|---|
| Niben101Scf04634g00004 | UP | Cellulose synthase A catalytic subunit 6 [UDP-forming] |
| Niben101Scf00573g01003 | UP | Asparagine synthetase [glutamine-hydrolyzing] |
| Niben101Scf11860g00013 | UP | Asparagine synthetase [glutamine-hydrolyzing] |
| Niben101Scf03307g06003 | UP | Inositol oxygenase 2 |
| Niben101Scf16156g00005 | UP | L-ascorbate oxidase homolog |
| Niben101Scf00449g06008 | DOWN | Heat shock protein 70 |
| Niben101Scf06684g01005 | DOWN | Heat shock protein 83 |
| Niben101Scf02389g00013 | DOWN | Protein of unknown function |

**Table S8. Shared DEGs between the P35S_A and PA_A lines.**

| Gene ID | Gene Expression | Annotation |
|---|---|---|
| Niben101Scf02363g00015 | UP | Beta-galactosidase |
| Niben101Scf10213g00009 | UP | Beta-galactosidase |
| Niben101Scf03253g02010 | UP | Glutamine--fructose-6-phosphate aminotransferase |
| Niben101Scf06374g00021 | UP | Protein PAT1 homolog 1 |
| Niben101Scf06570g05003 | UP | Pollen Ole e 1 allergen protein |
| Niben101Scf01785g04007 | UP | Two-component response regulator ARR9 |
| Niben101Ctg13782g00003 | UP | Probable xyloglucan endotransglucosylase/hydrolase protein 23 |
| Niben101Scf03326g01009 | UP | Cellulose synthase-like protein D3 |
| Niben101Scf04094g02006 | UP | Cellulose synthase A catalytic subunit 6 [UDP-forming] |
| Niben101Scf04634g00004 | UP | Cellulose synthase A catalytic subunit 6 [UDP-forming] |
| Niben101Scf11860g00013 | UP | Asparagine synthetase [glutamine-hydrolyzing] |
| Niben101Scf00573g01003 | UP | Asparagine synthetase [glutamine-hydrolyzing] |
| Niben101Scf01719g08001 | UP | Pleiotropic drug resistance protein 1 |
| Niben101Scf06583g03009 | UP | Pleiotropic drug resistance protein 1 |
| Niben101Scf03619g05002 | UP | Polyphenol oxidase E, chloroplastic |
| Niben101Scf07069g01004 | UP | Calmodulin-binding protein 60 B |
| Niben101Scf06222g01010 | UP | Calmodulin-binding protein 60 C |
| Niben101Scf01934g02004 | UP | Glucan endo-1,3-beta-glucosidase, basic vacuolar isoform GLB |
| Niben101Scf02349g03001 | UP | Suberization-associated anionic peroxidase |
| Niben101Scf07253g02020 | UP | Probable polyamine oxidase 4 |
| Niben101Scf03307g06003 | UP | Inositol oxygenase 2 |
| Niben101Scf02581g04013 | UP | Respiratory burst oxidase homolog protein C |
| Niben101Scf10575g02003 | UP | Putative lipid-transfer protein DIR1 |
| Niben101Scf09044g01015 | UP | Osmotin-like protein |
| Niben101Scf05615g01004 | DOWN | Cytochrome P450 CYP72A219 |
| Niben101Scf00449g06008 | DOWN | mediator of RNA polymerase II transcription subunit 37e |
| Niben101Scf06684g01005 | DOWN | Heat shock protein 83 |
| Niben101Scf01084g05013 | DOWN | Protein of unknown function |

**Table S9. Shared DEGs between the P35S_B and PB_B lines.**

| Gene ID | Gene Expression | Annotation |
|---|---|---|
| Niben101Scf02363g00015 | UP | Beta-galactosidase |
| Niben101Scf10213g00009 | UP | Beta-galactosidase |
| Niben101Scf03253g02010 | UP | Glutamine--fructose-6-phosphate aminotransferase |
| Niben101Scf03015g06014 | UP | Ribulose bisphosphate carboxylase small chain 8B, chloroplastic |
| Niben101Scf02658g00012 | UP | SKP1-like protein 1A |
| Niben101Ctg13782g00003 | UP | Probable xyloglucan endotransglucosylase/hydrolase protein 23 |
| Niben101Scf00109g03008 | UP | Glycerophosphodiester phosphodiesterase GDPD2 |
| Niben101Scf03326g01009 | UP | Cellulose synthase-like protein D3 |
| Niben101Scf04634g00004 | UP | Cellulose synthase A catalytic subunit 6 [UDP-forming] |
| Niben101Scf03913g01045 | UP | Expansin-like A2 |
| Niben101Scf08047g01012 | UP | Expansin-like A1 |
| Niben101Scf00573g01003 | UP | Asparagine synthetase [glutamine-hydrolyzing] |
| Niben101Scf11860g00013 | UP | Asparagine synthetase [glutamine-hydrolyzing] |
| Niben101Scf02738g01016 | UP | Proline dehydrogenase 2, mitochondrial |
| Niben101Scf03307g06003 | UP | Inositol oxygenase 2 |
| Niben101Scf16156g00005 | UP | L-ascorbate oxidase homolog |
| Niben101Scf15666g00009 | UP | Ribosome-binding factor PSRP1, chloroplastic |
| Niben101Scf03822g00010 | UP | Protein EXORDIUM-like 5 |
| Niben101Scf10763g00008 | UP | Stem-specific protein TSJT1 |
| Niben101Scf04024g02024 | DOWN | MATE efflux family protein 7 |
| Niben101Scf00887g02002 | DOWN | Protein REVEILLE 7 |
| Niben101Scf01685g09002 | DOWN | Calcium-binding protein PBP1 |
| Niben101Scf05522g00002 | DOWN | Calcium-binding protein PBP1 |
| Niben101Scf00799g00011 | DOWN | NAC domain-containing protein 2 |
| Niben101Scf03028g01011 | DOWN | NAC domain-containing protein 2 |

| | | |
|---|---|---|
| Niben101Scf00802g00005 | DOWN | U-box domain-containing protein 19 |
| Niben101Scf04296g00008 | DOWN | U-box domain-containing protein 19 |
| Niben101Scf00163g22002 | DOWN | Ethylene-responsive transcription factor 5 |
| Niben101Scf00163g22004 | DOWN | Ethylene-responsive transcription factor 5 |
| Niben101Scf00454g03001 | DOWN | Ethylene-responsive transcription factor 5 |
| Niben101Scf00846g02001 | DOWN | Ethylene-responsive transcription factor RAP2-13 |
| Niben101Scf01752g05003 | DOWN | Ethylene-responsive transcription factor 3 |
| Niben101Scf23241g00003 | DOWN | Ethylene-responsive transcription factor 5 |
| Niben101Scf08546g03002 | DOWN | Ethylene-responsive transcription factor 5 |
| Niben101Scf02362g02014 | DOWN | Probable WRKY transcription factor 33 |
| Niben101Scf02430g03006 | DOWN | Probable WRKY transcription factor 33 |
| Niben101Scf06822g04004 | DOWN | Transcription factor MYC2 |
| Niben101Scf15224g00002 | DOWN | Transcription factor MYC2 |
| Niben101Ctg12683g00001 | DOWN | Zinc finger CCCH domain-containing protein 29 |
| Niben101Scf02286g01005 | DOWN | Zinc finger CCCH domain-containing protein 29 |
| Niben101Scf12797g00017 | DOWN | Zinc finger CCCH domain-containing protein 29 |
| Niben101Scf00080g05006 | DOWN | Zinc finger protein ZAT10 |
| Niben101Scf00449g06008 | DOWN | Heat shock protein 70 |
| Niben101Scf12868g00008 | DOWN | Heat shock cognate 70 kDa protein 2 |
| Niben101Scf06684g01005 | DOWN | Heat shock protein 83 |
| Niben101Scf04867g00001 | DOWN | Scarecrow-like protein 14 |
| Niben101Scf06996g02005 | DOWN | Scarecrow-like protein 11 |
| Niben101Scf08041g02010 | DOWN | E3 ubiquitin-protein ligase RING1 |
| Niben101Scf09678g00006 | DOWN | Serine/arginine-rich splicing factor SR45a |
| Niben101Scf01750g15003 | DOWN | Probable CCR4-associated factor 1 homolog 11 |
| Niben101Scf10855g08005 | DOWN | Probable CCR4-associated factor 1 homolog 11 |
| Niben101Scf00611g09003 | DOWN | Putative nuclease HARBI1 |
| Niben101Scf34484g00001 | DOWN | Putative nuclease HARBI1 |
| Niben101Scf05756g00005 | DOWN | Putative nuclease HARBI1 |
| Niben101Scf01109g03004 | DOWN | Putative nuclease HARBI1 |
| Niben101Scf19533g00007 | DOWN | Protein kinase 2B, chloroplastic |
| Niben101Scf02303g00022 | DOWN | Exocyst complex component EXO70A1 |
| Niben101Scf11689g03003 | DOWN | BTB/POZ domain-containing protein At5g41330 |
| Niben101Scf00712g16028 | UP | BTB/POZ domain-containing protein |
| Niben101Scf08804g02014 | DOWN | Nudix hydrolase 17, mitochondrial |
| Niben101Scf09225g04002 | DOWN | Beta-D-glucosyl crocetin beta-1,6-glucosyltransferase |
| Niben101Scf00573g00010 | DOWN | Probable protein phosphatase 2C 2 |
| Niben101Scf07040g00001 | UP | Probable protein phosphatase 2C 5 |

| | | |
|---|---|---|
| Niben101Scf06383g01007 | UP | Probable protein phosphatase 2C 5 |
| Niben101Scf01109g03005 | DOWN | Protein of unknown function |

**Table S10. Significant GO enrichment pathways of the genetically modified (GM) *N. benthamiana* lines.**

| Comparison group | GO pathway | GO ID | Gene Number | Corrected P-value |
|---|---|---|---|---|
| P35S_A vs WT | carbohydrate metabolic process | GO:0005975 | 13 | 0.027126 |
| | oxidoreductase activity, acting on peroxide as acceptor | GO:0016684 | 5 | 0.034676 |
| PA_A vs WT | structural constituent of cell wall | GO:0005199 | 6 | 8.70E-07 |
| | plant-type cell wall organization | GO:0009664 | 6 | 1.22E-05 |
| | plant-type cell wall organization or biogenesis | GO:0071669 | 6 | 1.22E-05 |
| | cellular glucan metabolic process | GO:0006073 | 7 | 0.0013551 |
| | glucan metabolic process | GO:0044042 | 7 | 0.0013551 |
| | cell wall organization | GO:0071555 | 6 | 0.0046685 |
| | carbohydrate metabolic process | GO:0005975 | 19 | 0.0046685 |
| | external encapsulating structure organization | GO:0045229 | 6 | 0.0058503 |
| | cellular carbohydrate metabolic process | GO:0044262 | 11 | 0.0094167 |
| | cellulose synthase activity | GO:0016759 | 4 | 0.020978 |
| | cellulose synthase (UDP-forming) activity | GO:0016760 | 4 | 0.020978 |
| | cell wall organization or biogenesis | GO:0071554 | 6 | 0.032003 |
| | transferase activity, transferring hexosyl groups | GO:0016758 | 9 | 0.03418 |
| | cellulose biosynthetic process | GO:0030244 | 4 | 0.03418 |
| | cellulose metabolic process | GO:0030243 | 4 | 0.036399 |
| | cellular polysaccharide metabolic process | GO:0044264 | 9 | 0.036399 |
| PB_B vs WT | carbohydrate metabolic process | GO:0005975 | 29 | 0.00065519 |
| PA_A vs P35S_A | structural constituent of cell wall | GO:0005199 | 3 | 0.00020834 |
| | plant-type cell wall organization | GO:0009664 | 3 | 0.00036911 |
| | plant-type cell wall organization or biogenesis | GO:0071669 | 3 | 0.00036911 |
| | cell wall organization | GO:0071555 | 3 | 0.0091721 |
| | external encapsulating structure organization | GO:0045229 | 3 | 0.0091721 |
| | cell wall organization or biogenesis | GO:0071554 | 3 | 0.024512 |
| PB_B vs P35S_B | structural constituent of cell wall | GO:0005199 | 3 | 0.0091491 |
| | plant-type cell wall organization | GO:0009664 | 3 | 0.016974 |
| | plant-type cell wall organization or biogenesis | GO:0071669 | 3 | 0.016974 |
| PA_A vs PB_B | structural constituent of cell wall | GO:0005199 | 7 | 2.99E-12 |
| | plant-type cell wall organization | GO:0009664 | 7 | 9.95E-09 |
| | plant-type cell wall organization or biogenesis | GO:0071669 | 7 | 9.95E-09 |

| | | | |
|---|---|---|---|
| cell wall organization | GO:0071555 | 7 | 5.44E-05 |
| cell wall organization or biogenesis | GO:0071554 | 8 | 5.44E-05 |
| external encapsulating structure organization | GO:0045229 | 7 | 5.86E-05 |
| peroxidase activity | GO:0004601 | 6 | 0.0035078 |
| oxidoreductase activity, acting on peroxide as acceptor | GO:0016684 | 6 | 0.0042536 |
| hydrolase activity, hydrolyzing O-glycosyl compounds | GO:0004553 | 9 | 0.016781 |
| antioxidant activity | GO:0016209 | 6 | 0.020492 |
| hydrolase activity, acting on glycosyl bonds | GO:0016798 | 9 | 0.022597 |
| response to oxidative stress | GO:0006979 | 5 | 0.022628 |
| beta-galactosidase complex | GO:0009341 | 3 | 0.02616 |
| beta-galactosidase activity | GO:0004565 | 3 | 0.02616 |
| galactosidase activity | GO:0015925 | 3 | 0.02616 |

**Table S11. Significant KEGG enrichment pathways of the GM *N. benthamiana* lines.**

| Comparison Group | KEGG Pathway | KEGG ID | Gene Number | Corrected P-value |
|---|---|---|---|---|
| P35S_A vs WT | Alanine, aspartate and glutamate metabolism | sly00250 | 4 | 0.00130653 |
| P35S_B vs WT | Alanine, aspartate and glutamate metabolism | sly00250 | 3 | 0.03924253 |
| PA_A vs WT | Alanine, aspartate and glutamate metabolism | sly00250 | 4 | 0.01021507 |
| PA_A vs PB_B | Glutathione metabolism | sly00480 | 6 | 0.00200214 |
| | Alanine, aspartate and glutamate metabolism | sly00250 | 4 | 0.00477255 |
| | Phenylalanine metabolism | sly00360 | 5 | 0.02537974 |
| | Biosynthesis of secondary metabolites | sly01110 | 14 | 0.03550397 |
| | Phenylpropanoid biosynthesis | sly00940 | 5 | 0.0378198 |
